# MicroRNA signature and integrative omics analyses define prognostic clusters and key pathways driving prognosis in patients with neuroendocrine neoplasms

**DOI:** 10.1002/1878-0261.13393

**Published:** 2023-03-05

**Authors:** Beatriz Soldevilla, Alberto Lens‐Pardo, Paula Espinosa‐Olarte, Carlos Carretero‐Puche, Sonia Molina‐Pinelo, Carlos Robles, Marta Benavent, Lourdes Gomez‐Izquierdo, Marta Fierro‐Fernández, Patricia Morales‐Burgo, Paula Jimenez‐Fonseca, Beatriz Anton‐Pascual, Yolanda Rodriguez‐Gil, Ana Teijo‐Quintans, Anna La Salvia, Beatriz Rubio‐Cuesta, Maria C. Riesco‐Martínez, Rocio Garcia‐Carbonero

**Affiliations:** ^1^ Centro de Oncología Experimental, Grupo de Investigación en Tumores Gastrointestinales y Neuroendocrinos Instituto de Investigación Sanitaria Hospital 12 de Octubre (imas12) Madrid Spain; ^2^ Centro Nacional de Investigaciones Oncológicas (CNIO) Madrid Spain; ^3^ Oncology Department Hospital Universitario 12 de Octubre Madrid Spain; ^4^ Hospital Universitario Virgen del Rocío, IBIS Sevilla Spain; ^5^ Instituto de Biomedicina de Sevilla (IBiS) (HUVR, CSIC, Universidad de Sevilla) Spain; ^6^ Centro de Biología Molecular Severo Ochoa (CSIC‐UAM) Madrid Spain; ^7^ Hospital Universitario Central de Asturias Oviedo Spain; ^8^ Pathology Department Hospital 12 de Octubre Madrid Spain; ^9^ Universidad Complutense de Madrid (UCM) Spain

**Keywords:** miRNAs, NEN biology, NEN patients, prognostic impact

## Abstract

Neuroendocrine neoplasms (NENs) are mutationally quiet (low number of mutations/Mb), and epigenetic mechanisms drive their development and progression. We aimed at comprehensively characterising the microRNA (miRNA) profile of NENs, and exploring downstream targets and their epigenetic modulation. In total, 84 cancer‐related miRNAs were analysed in 85 NEN samples from lung and gastroenteropancreatic (GEP) origin, and their prognostic value was evaluated by univariate and multivariate models. Transcriptomics (*N* = 63) and methylomics (*N* = 30) were performed to predict miRNA target genes, signalling pathways and regulatory CpG sites. Findings were validated in The Cancer Genome Atlas cohorts and in NEN cell lines. We identified a signature of eight miRNAs that stratified patients in three prognostic groups (5‐year survival of 80%, 66% and 36%). Expression of the eight‐miRNA gene signature correlated with 71 target genes involved in PI3K–Akt and TNFα–NF‐kB signalling. Of these, 28 were associated with survival and validated *in silico* and *in vitro*. Finally, we identified five CpG sites involved in the epigenetic regulation of these eight miRNAs. In brief, we identified an 8‐miRNA signature able to predict survival of patients with GEP and lung NENs, and identified genes and regulatory mechanisms driving prognosis in NEN patients.

## Graphical abstract

The comprehensive characterisation of microRNA (miRNA) profiles of neuroendocrine neoplasms has identified an eight‐miRNA signature able to stratify patients into three prognostic groups. Transcriptomic data have shown that these miRNAs are associated with genes involved in PI3K–Akt and TNFα–NF‐kB signalling. Finally, we have identified five CpG sites involved in the epigenetic regulation of these eight miRNAs.

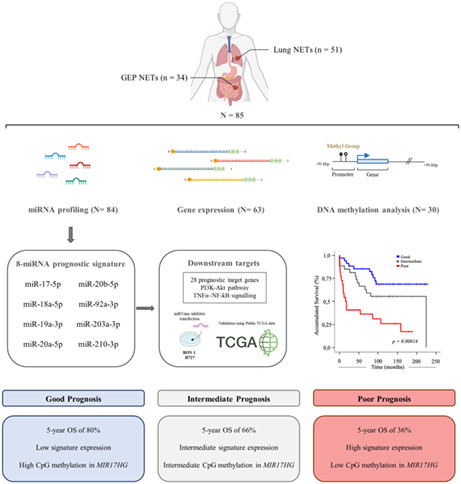

**Abbreviations**
*ACTB*
: actin beta
*AGFG2*
: ArfGAP with FG repeats 2
*ALG2*
: ALG2 alpha‐1,3/1,6‐mannosyltransferase
*ARID1A*
: AT‐rich interaction domain 1A
*ATRX*
: ATRX chromatin remodeler
*CDKN1B*
: cyclin‐dependent kinase inhibitor 1B
cDNA: complementary deoxyribonucleic acid
*CRY2*
: cryptochrome circadian regulator 2
Cts: cycle thresholds
*DAXX*
: death domain‐associated protein
FDR: false discovery rate
FFPE: formalin‐fixed paraffin‐embedded
G1: grade 1
G2: grade 2
G3: grade 3
GEP: gastroenteropancreatic
*HIF1A*
: hypoxia‐inducible factor 1 subunit alpha
*MEN1*
: menin 1
*MINK1*
: Misshapen‐like kinase 1
*MIR17HG*
: MiR‐17‐92a‐1 cluster host gene
miRNA: microRNA
mRNA: messenger ribonucleic acid
NECs: poorly differentiated neuroendocrine carcinomas
NENs: neuroendocrine neoplasms
NETs: well‐differentiated neuroendocrine tumours
ORA: overrepresentation analysis
OS: overall survival
qRT‐PCR: quantitative real‐time polymerase chain reaction
*REEP3*
: receptor accessory protein 3
*RHOB*
: Ras homolog family member B
*RNU6B*
: RNA, U6 small nuclear 6, pseudogene
RPKM: reads per kilobase million
RT: reverse transcription
SEER: surveillance, epidemiology and end results
*SNORD61*
: small nucleolar RNA, C/D Box 61
*SNORD68*
: small nucleolar RNA, C/D Box 68
*SNORD95*
: small nucleolar RNA, C/D Box 95
*SNORD96A*
: small nucleolar RNA, C/D Box 96A
TCGA: The Cancer Genome Atlas
TSS: transcription start sites
*UTR*
: untranslated region
*VEGF*
: vascular endothelial growth factor A

## 1 Introduction

Neuroendocrine neoplasms (NENs) are a heterogeneous family of malignancies that arise from the diffuse neuroendocrine system and have thus a wide anatomic distribution [1]. Although they can arise in virtually any organ, most common primary tumour sites are the lungs (25%) and the digestive tract (~ 60%) [2]. Based on their morphology, NENs are classified into two main subgroups: the generally more indolent, well‐differentiated neuroendocrine tumours (NETs) that account for ~ 80% of NENs and are subclassified by their proliferative index in grade 1 (Ki‐67 < 3%), 2 (Ki‐67 3–20%) or 3 (Ki‐67 > 20%), and the more aggressive, poorly differentiated neuroendocrine carcinomas (NECs) that always present high proliferative rates (grade 3 or Ki‐67 > 20%) and are associated with a very poor prognosis [3, 4, 5]. Historically, NENs are considered a rare cancer type (< 5 new cases diagnosed annually per 100 000 habitants) [2, 6], but their incidence has increased 6.4‐fold over the past four decades (from 1.09 (1973) to 6.98 (2012) per 100 000 according to SEER data [2]). The 5‐year overall survival (OS) rate for all NENs is about 50% (64% for NETs, 12% for NECs), but survival notably varies by tumour differentiation, grade, stage, primary tumour site, age at diagnosis and even year of diagnosis. Indeed, median survival ranges widely from 16.2 years for G1 (grade 1) to < 1 year for G3 (grade 3) NENs but has significantly improved over time, reflecting earlier diagnosis and therapeutic improvements [6].

The low incidence, anatomical dispersion, and molecular and clinical heterogeneity of NENs have hindered an in‐depth characterisation of the molecular landscape of these tumours. These neoplasia, and particularly NETs, are mutationally quiet, harbouring relatively few somatic mutations as compared to other malignancies [7, 8]. Studies in sporadic NENs have reported few recurrently mutated genes: *MEN1* (20%) and *ARID1A* (8%) in lung NETs [8], *MEN1* (40%) and *ATRX/DAXX* (35%) in pancreatic NETs [9] and *CDKN1B* (10%) in small intestine NETs [10]. This would suggest that there are other alternative mechanisms implicated in the development and progression of these tumours. Consistent with this, several recent reports suggested that epigenetic changes may play a key role in the pathogenesis of NENs [10, 11, 12]. Nevertheless, a better characterisation of these nonmutational alterations is required to improve our understanding of the molecular mechanisms involved in the biological and clinical behaviour of NENs. Alterations in epigenetic regulators such as microRNAs (miRNAs) or DNA methylation could be relevant in this context as they can modulate gene expression and, consequently, specific oncogenic or tumour suppressive pathways. Moreover, as these changes are reversible, epigenetic modulation could potentially be exploited for therapeutic purposes.

In this context, the aim of this study was to comprehensively characterise the miRNA profile of NENs and to explore, through integrative omics analyses, downstream targets and epigenetic modulation to identify key pathways involved in NEN biological and clinical behaviour.

## 2 Materials and methods

### 2.1 Patients and samples

The study population included 85 patients with G1‐G3 NENs of gastroenteropancreatic (GEP; *N* = 34) and lung (*N* = 51) origin diagnosed between 2000 and 2015 (small cell neuroendocrine carcinoma of lung origin was excluded) in the Hospital Universitario Virgen del Rocío (HUVR) and Hospital Universitario Central de Asturias (HUCA). Archival formalin‐fixed paraffin‐embedded (FFPE) tumour and nontumour tissue samples from each patient were retrospectively collected and assessed by expert NEN pathologists prior to molecular analysis.

The study was carried out in accordance with the Declaration of Helsinki and approved, together with the protocols, by the Hospital 12 de Octubre ethics committee (Committee Register Number: 18/449). All patients provided written informed consent prior to the study entry.

### 2.2 Cell culture

The BON‐1 cell line (RRID: CVCL_3985; kindly provided by J. Castaño, Instituto Maimonides de Investigación Biomédica, Spain) and the H727 cell line (RRID:CVCL_1584; Sigma‐Aldrich, St. Louis, MO, USA) were cultured in 1 : 1 DMEM/F12K (Sigma‐Aldrich and Thermo Scientific, Rockford, IL, USA) and RPMI 1640 (Sigma‐Aldrich) medium, respectively, supplemented with 10% foetal bovine serum (TICO Europe, Amstelveen, the Netherlands) at 37 °C in 5% CO_2_.

For transient transfections, 2.5 × 10^5^ BON‐1 and H727 cells were, respectively, seeded in MW6 plates to reach 60–80% confluence. After 24 h, cells were transfected for 48 h with 60 nm of miRVana inhibitors (MH12412 and MH10649 for mirR‐17‐5p and miR‐19a‐3p, respectively (Thermo Scientific)) using Lipofectamine RNAiMAX Transfection Reagent (Thermo Scientific). An equal concentration of miRVana Inhibitor Negative control #1 (4464076; Thermo Scientific) was used in all experiments. BON‐1 cell line was authenticated using short tandem repeat (STR) profiling (3/12/2020). H727 cell line was obtained from ATCC (01/08/2019), confirming its authenticity. All experiments with BON‐1 and H727 cell lines were performed after check the absence of mycoplasma contamination (MycoAlert™ Mycoplasma Detection Kit; Lonza, Basilea, Switzerland).

### 2.3 Nucleic acid extraction

Total DNA and RNA were extracted using standard methods (QIAamp DNA FFPE Tissue Kit (QIAGEN, Valencia, CA, USA) and Recover All TM Total Nucleic Acid Isolation Ambition Kit (Applied Biosystems, Foster City, CA, USA)) according to the manufacturers' instructions. Total RNA was extracted from cell lines using the miRNeasy Mini Kit (QIAGEN) according to the manufacturer's instructions. DNA and RNA integrity, purity and concentration were assessed with the Qubit® Fluorimeter (Life Technologies, Carlsbad, CA, USA) or Nanodrop 1000 (NanoDrop, Wilmington, DE, USA) instruments, respectively.

### 2.4 Analysis of miRNA expression profiles

Quantitative Real‐Time PCR (qRT‐PCR) was performed to determine the expression levels of mature human miRNAs. Complementary DNA (cDNA) was obtained by reverse transcription (RT) of extracted RNA from tumour and nontumour patient samples using the cDNA miScript II Reverse Transcription kit (QIAGEN). Expression levels of miRNAs were determined using the Human Cancer Pathway Finder miScript miRNA PCR Array (MIHS‐102ZE; QIAGEN), which included 84 of the most relevant cancer‐related human miRNAs. Reaction conditions for amplification were as follows: first step of 95 °C 15 min, then 40 cycles of three‐step 94 °C 15 s, 55 °C 30 s and 70 °C 30 s. For miRNA expression analysis, we have used nontumour paired tissues to normalise the miRNA data for each tumour sample. Expression of miRNAs for each NEN patient was assessed by a relative quantification approach, in which the amount of each miRNA is expressed in relation to the geometric average of five reference housekeeping genes (small nucleolar RNAs: *SNORD61*, *SNORD68*, *SNORD95*, *SNORD96A*, *RNU6B*) using the $2^{-\Delta \Delta C_{t}}$ method [13]: Δ*C*_t_ = *C*_t_ (miRNA) − *C*_t_ (average of the housekeeping genes); ΔΔ*C*_t_ = Δ*C*_t_ (tumour) − Δ*C*_t_ (nontumour); $2^{-\Delta \Delta C_{t}}$ = ‘fold change’: relative expression of the miRNA in tumour sample with respect to the normal tissue. Expression of eight selected miRNAs (miR‐17‐5p, miR‐18a‐5p, miR‐19a‐3p, miR‐20a‐5p, miR‐20b‐5p, miR‐92a‐3p, miR‐203a‐3p and miR‐210‐3p; reference numbers 002308, 002422, 000395, 000580, 001014, 000431, 000507, and 000512, respectively; Thermo Scientific) was also quantified in 40 NENs with available tumour and nontumour samples using TaqMan qPCR as an alternative method (Table S1). Geometric average of SNORD95 and SNORD61 (reference numbers Hs03464473_s1 and Hs03298134_s1, respectively; Thermo Scientific) was used to normalise the data. In all, 10 ng of total RNA was reverse transcribed using the TaqMan miRNA reverse transcription kit in a total volume of 15 μL, according to the manufacturer's protocol. Thereafter, 0.7 μL of cDNA was used for TaqMan MicroRNA Assays. The reactions were incubated at 95 °C for 10 min, followed by 40 cycles of two‐step 95 °C 15 s and 60 °C 1 min. All reactions were performed in an Applied Biosystems ViiA 7™ Real‐Time PCR instrument (BioRad, Hercules, CA, USA).

### 2.5 Gene expression and DNA methylation profiling

Clariom S Human microarrays (Affimetrix, Santa Clara, CA, USA) representing more than 23 000 unique transcripts were performed in all tumour samples with sufficient remaining RNA (*N* = 63). After assessing the RNA integrity, labelling and hybridisation were performed according to Affimetrix protocols. Briefly, 50 ng of total RNA was amplified and labelled using the Pico reagent kit (Affimetrix) and then hybridised to Clariom S Human microarrays (Affimetrix). Washing and scanning were performed using the Affymetrix GeneChip System (GeneChip Hybridization Oven 645, GeneChip Fluidics Station 450 and GeneChip Scanner 7G).

### 2.6 DNA methylation profiling

Total DNA was extracted from paired FFPE tumour (*N* = 30) and nontumour (*N* = 10) tissue samples using the QIAamp DNA FFPE Tissue Kit (QIAGEN) according to the manufacturer's recommendations. DNA purity and concentration were assessed with the Qubit® Fluorimeter (Life Technologies). Quality control and normalisation were performed with the Infinium HD FFPE QC assay (Illumina, San Diego, CA, USA) kit. Samples that passed the quality control were subjected to bisulfite conversion using 250 ng of DNA and the EZ‐96 DNA Methylation Kit (Zymo Research, Irvine, CA, USA). Converted DNA was processed with the Infinium HD FFPE Restore Protocol (Illumina), and the ZR‐96 DNA Clean & Concentrator‐5 (Deep Well) (Zymo Research) kit to restore FFPE DNA. Methylation analysis was performed using Infinium MethylationEPIC BeadChip (Illumina), which allows extensive coverage of CpG islands and gene enhancers. Fluorescence detection and scanning were performed with the iScan System (Illumina) platform.

The obtained raw data files (.IDAT) were processed and filtered using the ChAMP pipeline available as a r package. The beta‐values obtained were transformed into *M*‐values to normalise distribution of the data. Methylation levels of CpG sites (beta‐values) were obtained for each sample and were compared between clusters defined by miRNAs expression in tumour and nontumour samples.

### 2.7 qRT‐PCR of selected miRNAs and target genes

MiR‐17‐5p and miR‐19a‐3p expressions in transiently transfected cell lines were assessed as described in the analysis of miRNA expression profiles. Selected miRNA target genes were analysed in transiently transfected cell lines by RT of 1000 ng of RNA using the High‐Capacity cDNA Reverse Transcription Kit (Thermo Scientific) according to the manufacturer's instructions. cDNA was amplified using TaqMan Universal PCR mix (Thermo Scientific) and *ALG2* (ref. Hs00263798_m1), *RHOB* (ref. Hs05051455_s1), *REEP3* (ref. Hs00416535_m1), *CRY2* (ref. Hs00901393_m1), *AGFG2* (ref. Hs00968746_m1) and *MINK1* (ref. Hs01093259_m1). *ACTB* (ID: Hs01060665_g1) was used as a reference gene to normalise cycle thresholds (*C*_t_s). Relative miRNA/mRNA expression levels were determined using the $2^{-\Delta \Delta C_{t}}$ method [13]. All reactions were performed in an Applied Biosystems ViiA 7™ Real‐Time PCR machine (BioRad).

### 2.8 Statistical analysis

Descriptive statistics were used to characterise most relevant clinical parameters. The distribution of quantitative variables among study groups was evaluated by parametric (Student's *t*‐test or ANOVA) and nonparametric (Kruskall–Wallis or Mann–Whitney) tests, as appropriate. OS was calculated as the time elapsed from the date of diagnosis to the date of death from any cause or last follow‐up of living patients. The Kaplan–Meier method was used to estimate time‐dependent variables (OS), and differences observed among patient subgroups were assessed by the log‐rank test. The prognostic value of all miRNAs was analysed by a Cox univariate regression model considering their expression as a continuous variable. *P*‐values were corrected for multiple testing by the false discovery rate (FDR) method, considering FDR ≤ 0.05 to indicate significance. Finally, a Cox multivariate proportional hazards model was used to further assess the prognostic value of selected miRNAs adjusted for other potentially confounding variables, such as gender, age, grade, TNM stage and primary tumour site (GEP or lung).

Spearman correlation analyses between selected miRNAs and *in silico* predicted target genes were performed. In addition, Cox regression analyses were performed to interrogate the role of significantly correlated target genes in OS (see Section 2.9). Correlations between miRNAs and CpG sites at regulatory regions (−50 kbp upstream from transcription start sites (TSS)) [14] were also assessed using Spearman correlations, with the Benjamini–Hochberg method used to adjust for multiple testing. MiRNA‐CpG sites with an FDR < 0.05 were considered significant. These CpG sites were further explored, and differences between tumour and normal samples, and between prognostic clusters were assessed with Student's *t*‐tests and ANOVA tests, respectively. *P*‐values < 0.05 were considered significant. All statistical analyses were performed using spss 21.0 (IBM) and r software (v4.1.2).

### 2.9 Bioinformatic analysis

*In silico* predicted targets for the eight miRNAs significantly associated with patient OS were identified using target genes consistently predicted in all three different databases explored: TargetScan Release 7.2 [15], miRDB [16] and Diana‐Tarbase v7.0 [17].

A heatmap showing the Spearman correlation coefficients among the 84 cancer‐related miRNAs expressions was created using the morpheus software (https://software.broadinstitute.org/morpheus/). Rows and columns of the heatmap were hierarchically clustered with 1—Spearman rank correlation as distance. On the contrary, a heatmap showing the expression levels of the eight miRNAs and the prognostic groups was obtained by complexheatmap [18] of the r software package using the UPGMA hierarchical clustering algorithm with Euclidean distance over the standardised Log (miRNA expression + 1).

An overrepresentation analysis (ORA) of the 71 significantly correlated target genes in Enrichr (https://maayanlab.cloud/Enrichr/) [19] was performed using KEGG [20] and MSigDB Hallmark 2020 [21] gene sets. Gene sets with an FDR < 0.25 were considered statistically significant. The functional network analysis was performed on String (https://string‐db.org/) [22] using 28 gene candidates as input selecting only for medium or high confidence interactions (*X* > 0.4).

### 2.10 Public database analysis

Normalised gene expression and miRNAs data from The Cancer Genome Atlas (TCGA) cohorts (Ovarian Serous Cystadenocarcinoma (TCGA, Nature 2011); Breast Invasive Carcinoma (TCGA, Nature 2012); Glioblastoma (TCGA, Nature 2008); Kidney Renal Clear Cell Carcinoma (TCGA, Nature 2013); Lung Squamous Cell Carcinoma (TCGA, Nature 2012); Colorectal Adenocarcinoma (TCGA, Nature 2012); and Pediatric Rhabdoid Tumor (TARGET, 2018)) were analysed using cBioPortal for Cancer Genomics (https://www.cbioportal.org/) [23]. The expression of selected genes was evaluated using *z*‐scores of either log RNA‐seq RPKM (Reads Per Kilobase Million), log RNA‐seq V2 RSEM or log microarray. Accordingly, miRNA expression was evaluated using *z*‐scores obtained from microarray expression data. Pearson's correlation analysis was assessed for all miRNA‐target gene pairs of interest. Values of *P* < 0.05 were considered significant. Only samples with both miRNA and mRNA expression data were included in the analysis for each TCGA study.

Similarly, normalised gene expression and DNA methylation data were also retrieved and analysed from the cBioPortal for Cancer Genomics (https://www.cbioportal.org/) [23]. The following TCGA studies were selected: Breast Invasive Carcinoma (TCGA, Firehose Legacy); Lung Adenocarcinoma (TCGA, Firehose Legacy); Acute Myeloid Leukemia (TCGA, Firehose Legacy); Esophageal Carcinoma (TCGA, Firehose Legacy); Kidney Renal Clear Cell Carcinoma (TCGA, Firehose Legacy); Lung Squamous Cell Carcinoma (TCGA, Firehose Legacy); Ovarian Serous Cystadenocarcinoma (TCGA, Firehose Legacy); and Glioblastoma Multiforme (TCGA, Firehose Legacy). *MIR17HG* expression was evaluated using *z*‐scores of log RNA‐seq V2 RSEM. Pearson's correlation analysis between *MIR17HG* gene expression and DNA methylation was assessed for each TCGA study. Values of *P* < 0.05 were considered statistically significant. Only samples with both *MIR17HG* expression and DNA methylation data were included in the analysis.

## 3 Results

### 3.1 Characteristics of the study population

The main clinical and pathological features of the study population are summarised in Table S1. With a median follow‐up of 131 months (range 26–229 months), 41 patients (48.2%) have died. The median OS of the study population was 91 months (range 0–229 months), with a 5‐year OS rate of 65%.

### 3.2 miRNA profiling identified eight miRNAs significantly associated with patient prognosis

**Fig. 1:**
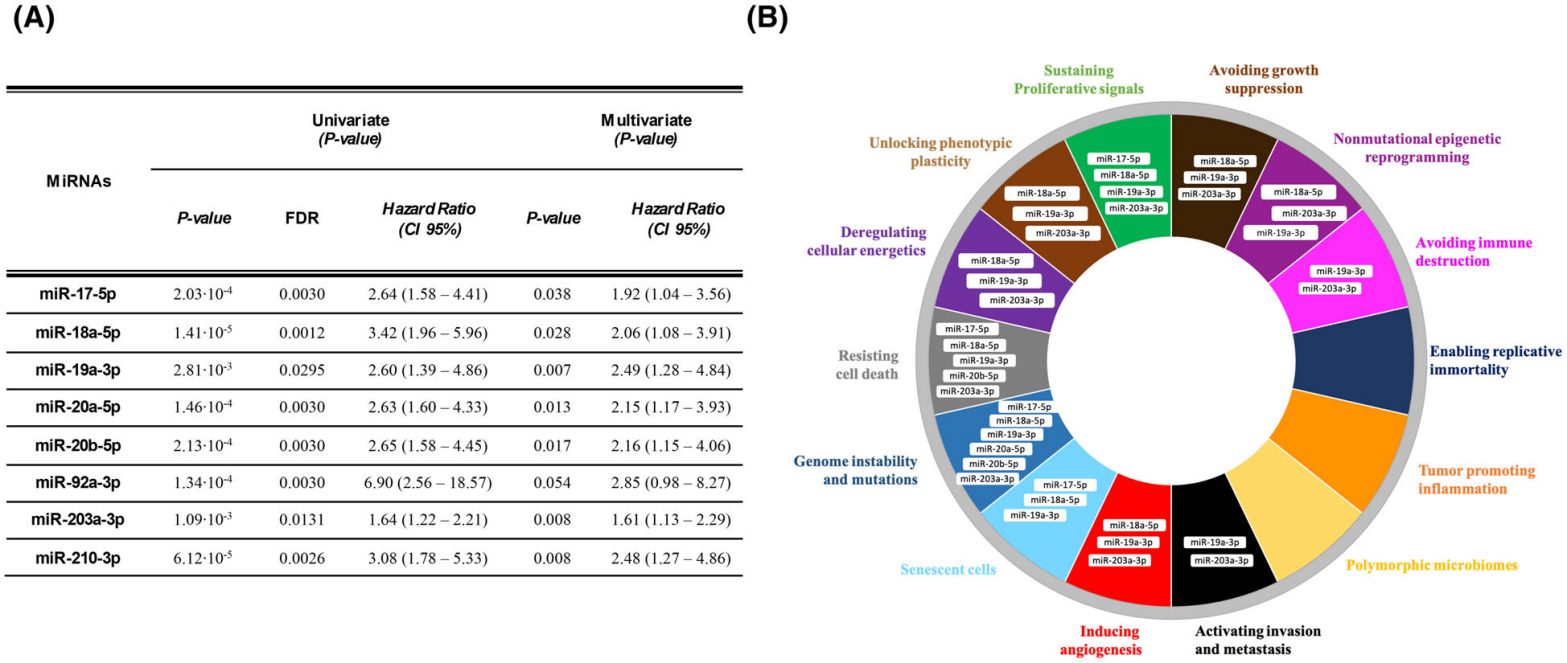
Eight miRNAs are associated with poor prognosis and key oncogenic pathways in NENs. (A) Prognostic impact of miRNAs in NENs. *P* < 0.05 was considered significant. CI, confidence interval. (B) The sector diagram represents the hallmarks of cancer [25] in which the eight prognostic miRNAs are involved based on an ORA of the 71 target genes that significantly correlate with these miRNAs.

To identify miRNAs most involved in the biology of NENs and in patients' clinical outcomes, we explored the association of 84 cancer‐related miRNAs with survival in 84 NEN patients (Table S1). The expression of 11 miRNAs was significantly associated with OS (FDR < 0.05). These miRNAs were miR‐17‐5p, miR‐18a‐5p, miR‐19a‐3p, miR20a‐5p miR‐20b‐5p, miR‐92a‐3p, miR‐203a‐3p, miR‐210‐3p, miR‐138‐5p, miR‐142‐5p and miR‐196a‐5p. Multivariate analyses demonstrated that overexpression of eight (miR‐17‐5p, miR‐18a‐5p, miR‐19a‐3p, miR20a‐5p miR‐20b‐5p, miR‐92a‐3p, miR‐203a‐3p and miR‐210‐3p) of these miRNAs was associated with a poorer prognosis, independent of other well‐established prognostic factors such as age, gender, tumour differentiation, stage and primary tumour location (Fig. 1A). Interestingly, a strong positive correlation among the expression levels of these eight miRNAs was observed across the whole cohort (Fig. S1), particularly for miR‐17‐5p, miR‐18a‐5p, miR‐19a‐3p, miR20a‐5p and miR‐92a‐3p. These five miRNAs belong to a cluster of miRNAs called miR‐17‐92 or *oncomiR‐1*, which has been described to be altered in other solid tumours. Moreover, miR‐20b‐5p is encoded by a miR‐17‐92 cluster gene paralogue named miR‐106a‐363 [24]. These results were validated in 40 NENs of our cohort (Table S1) using qRT‐PCR as an alternative method (Table S2, Fig. S2).

### 3.3 MiRNA and mRNA integrative expression analyses enabled the identification of key genes and pathways involved in NEN's biology

To further investigate which genes and pathways are regulated by the eight prognostic miRNAs, an integrative gene expression analysis of NEN samples (*N* = 63) was performed (Table S1). First, we performed correlation analysis between the miRNA expression and their *in silico* predicted target genes (*N* = 62) (Table S3; see Section 2). Seventy‐one of these target genes were found to be significantly correlated (*P* < 0.05) with their corresponding miRNA following integrative expression analyses performed on tumour samples from our study population; 41 of them were inversely correlated (*r* < 0), and 30 were directly associated (Table S4). ORA of these 71 target genes revealed involvement of the precited target genes in key signalling pathways including Hallmarks TNFα Sig. via NF‐kB and KEGG PI3K‐Akt signalling pathways, among others (FDR < 0.25) (Table S5). All of these dysregulated pathways are implicated in biological functions related to almost all hallmarks of cancer [25] (Fig. 1B).

We next interrogated the prognostic impact of the 71 target genes and found that 28 of them were significantly associated with OS (*P* < 0.05) (Table 1). The genes associated with poor prognosis were positively correlated with miRNA expression (*r* > 0) and those associated with better prognosis were negatively correlated with their corresponding miRNA (*r* < 0). Interestingly, a String network of these 28 prognostic genes revealed seven functionally related genes involved in mitotic spindle, G2/M checkpoint, apoptosis and cell cycle and proliferation (Fig. S3).

**Table 1:** List of predicted target genes of the 8‐miRNA signature with significant prognostic impact. Cox univariate regression model outcomes using OS and gene expression data of predicted target genes as continuous variables in 63 NENs are shown. Hazard ratios (HR) and *P*‐values are shown as well as the adjusted statistical significance (FDR). Twenty‐eight predicted target genes had significant prognostic impact (*P* < 0.05): 12 were associated with a poorer prognosis and 16 were associated with better outcome.

Gene	HR (CI 95%)	*P*‐value	FDR	MiRNA
*PRC1*	2.954 (1.97–4.42)	1 × 10^−7^	4.7 × 10^−4^	miR‐19a‐3p
*KIF23*	9.473 (3.44–26.05)	1.32 × 10^−5^	9.93 × 10^−3^	miR‐20b‐5p
*PON2*	0.019 (0.002–0.13)	7.83 × 10^−5^	3.024 × 10^−2^	miR‐20a‐5p
*CRY2*	0.158 (0.06–0.41)	1.291 × 10^−4^	3.932 × 10^−2^	miR‐17‐5p
*E2F3*	6.246 (2.44–15.97)	1.319 × 10^−4^	3.932 × 10^−2^	miR‐17‐5p
*HNRNPUL1*	5.628 (1.97–16.07)	1.253 × 10^−3^	ns	miR‐19a‐3p
*DBN1*	13.349 (2.75–64.75)	1.298 × 10^−3^	ns	miR‐19a‐3p
*ATP6V0E1*	0.302 (0.142–0.639)	1.738 × 10^−3^	ns	miR‐19a‐3p
*AGFG2*	0.269 (0.11–0.62)	2.067 × 10^−3^	ns	miR‐17‐5p
*CENPQ*	5.326 (1.81–15.64)	2.346 × 10^−3^	ns	miR‐17‐5p
*FAM199X*	0.067 (0.01–0.39)	2.8593 × 10^−3^	ns	miR‐20a‐5p
*E2F8*	3.398 (1.44–7.98)	5.0125 × 10^−3^	ns	miR‐19a‐3p
*ATXN7*	0.275 (0.10–0.70)	7.4595 × 10^−3^	ns	miR‐92a‐3p
*CCND2*	0.103 (0.04–0.54)	7.6525 × 10^−3^	ns	miR‐18a‐5p
*KPNA6*	0.342 (0.14–0.79)	1.244 × 10^−2^	ns	miR‐18a‐5p
*SAMD8*	0.243 (0.08–0.76)	1.505 × 10^−2^	ns	miR‐20b‐5p
*REEP3*	0.281 (0.10–0.78)	1.576 × 10^−2^	ns	miR‐19a‐3p
*AMFR*	0.128 (0.03–0.69)	1.704 × 10^−2^	ns	miR‐203a‐3p
*PKIA*	3.569 (1.24–10.25)	1.808 × 10^−2^	ns	miR‐20a‐5p
*MINK1*	0.215 (0.06–0.77)	1.828 × 10^−2^	ns	miR‐17‐5p
*ALG2*	0.368 (0.16–0.86)	2.121 × 10^−2^	ns	miR‐19a‐3p
*C6orf120*	9.718 (1.37–68‐53)	2.251 × 10^−2^	ns	miR‐17‐5p
*PIGS*	0.298 (0.10–0.88)	2.933 × 10^−2^	ns	miR‐19a‐3p
*DUT*	6.334 (1.20–33.34)	2.939 × 10^−2^	ns	miR‐19a‐3p
*XRN2*	3.374 (1.12–10.16)	3.066 × 10^−2^	ns	miR‐203a‐3p
*SCAMP5*	0.291 (0.09–0.92)	3.584 × 10^−2^	ns	miR‐20a‐5p
*ZNF217*	3.313 (1.04–10.55)	4.293 × 10^−2^	ns	miR‐19a‐3p
*RHOB*	0.395 (0.16–0.97)	4.293 × 10^−2^	ns	miR‐19a‐3p

### 3.4 *In vitro* and *in silico* validation of target genes of the eight prognostic miRNAs

**Fig. 2:**
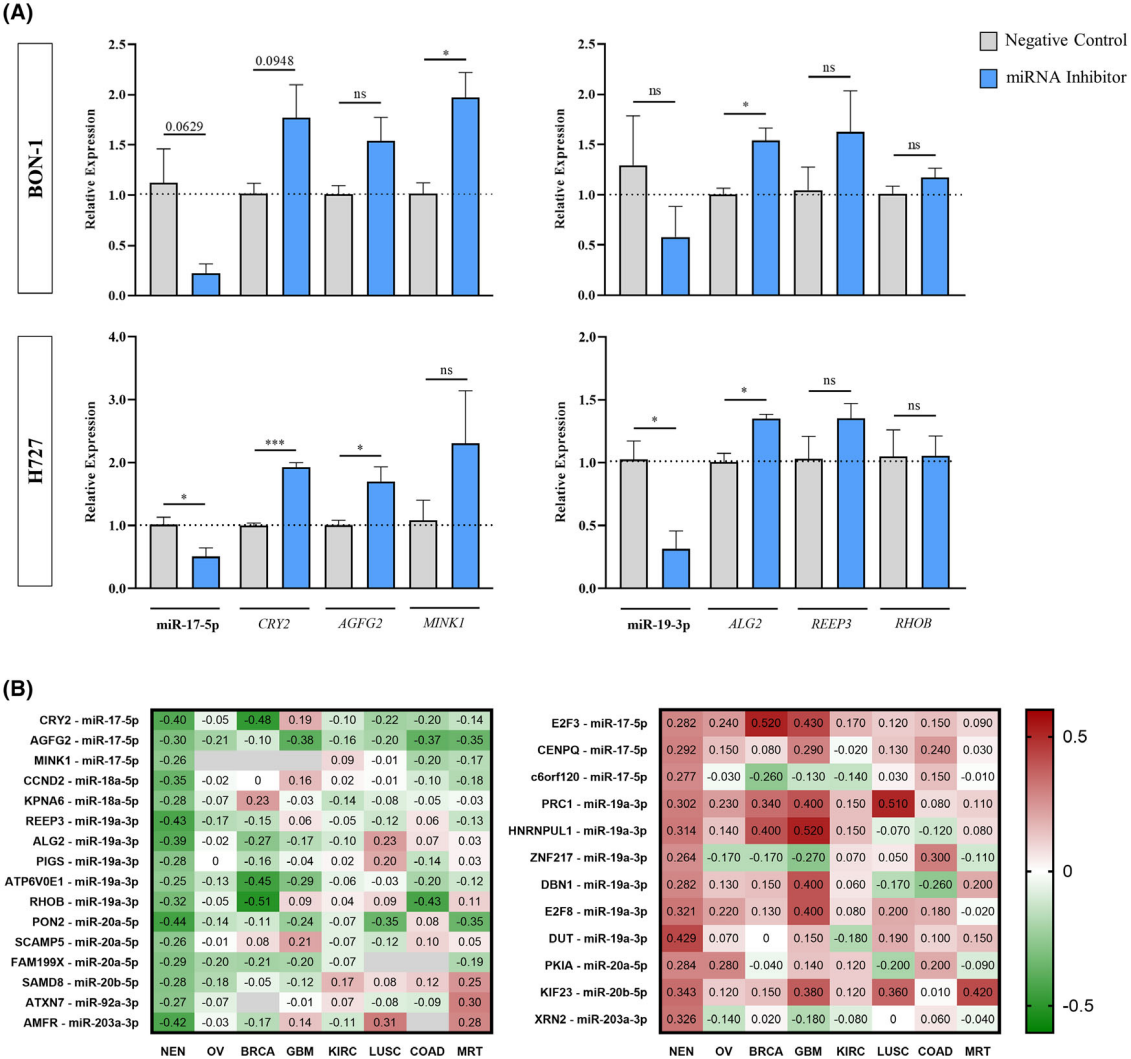
Validation of target genes of the eight prognostic miRNAs. (A) MiR‐17‐5p and miR‐19a‐3p regulate the expression of their predicted target genes in NENs cell lines. MiR‐17‐5p inhibitor was transfected for 48 into H727 and BON‐1 cell lines using lipofectamine (*n* = 3). Treated BON‐1 and H727 cells showed a reduced expression of miR‐17‐5p at 48 h upon transfection. Accordingly, expression of candidate target genes *CRY2* and *AGFG2* was significantly increased in H727 cells and expression of *MINK1* was significantly increased in BON‐1 cells. A nonsignificant increase of *MINK1* and of *CRY2* and *AGFG2* was observed in H727 and BON‐1 cell lines, respectively. Similarly, miR‐19a‐3p inhibitor was transfected for 48 h into H727 and BON‐1 cell lines using lipofectamine (*n* = 3). Treated BON‐1 and H727 cells showed a reduced expression of miR‐19a‐3p at 48 h upon transfection. Accordingly, the expression of candidate target genes *ALG2* and *REEP3* was increased in the BON‐1 and H727 cell line, although it did not reach statistical significance for *REEP3*. No differences were observed in *RHOB*. MiRNA or target gene expression levels are expressed as $2^{-\Delta \Delta C_{t}}$ on the *y*‐axis using the negative control at each time as reference. The dotted line represents the NC (Negative Control) value. Mean ± SEM (Standard Error of the Mean) is shown. Student's *t*‐tests between the negative control and inhibitor‐transfected cells at each time point were performed to evaluate differences, with *P*‐values <0.05 considered statistically significant (*P < 0.05; ***P < 0.001; ns, nonsignificant). (B) Correlations between miRNAs and target gene expression were validated *in silico* in different solid tumours. We performed Pearson's correlation analyses for each miRNA/target gene pair in seven solid tumour TCGA cohorts. Heatmaps represent the miRNA‐target gene coefficient of our NEN cohort and of the seven TCGA cohorts. Inversely (left panel) and directly (right panel) correlated miRNA‐target genes are shown in the two heatmaps. The seven TCGA databases and our NEN cohort are shown in columns and miRNA‐target genes are shown in rows. Individual Pearson's correlation coefficient values are colour‐coded, ranging from red (positive correlation; max = 1) to green (negative correlation; min = −1). Overall, negatively correlated genes from our study were also negatively correlated in the TCGA samples (in green), and directly correlated genes from our study were also positively correlated in the TCGA (red). OV, ovarian serous cystadenocarcinoma; BRCA, breast invasive carcinoma; GBM, glioblastoma; KIRC, kidney renal clear cell carcinoma; LUSC, lung squamous cell carcinoma; COAD, colorectal adenocarcinoma; MRT, malignant rhabdoid tumor.

To further validate a direct correlation between the eight miRNAs and some of their target genes observed in patient samples, we explored target gene expression upon downregulation of miR‐17‐5p and miR‐19a‐3p in BON‐1 pancreatic and H727 lung NET cell lines. Basal expression levels of miR‐17‐5p and miR‐19a‐3p in NET cell lines were similar to those obtained in the good prognosis cluster of NENs, whereas a nonsignificant trend was observed towards higher miRNA levels in tumour samples of patients of the poor prognosis cluster (Fig. S4). As expected, inhibition of miR‐17‐5p in the H727 cell line significantly increased the expression of its targets *CRY2* and *AGFG2*. A nonsignificant increase trend was observed for *MINK1* (Fig. 2A). In BON‐1 cell lines, a significant increase of *MINK1* and a nonsignificant trend in *CRY2* and *AGFG2* was observed upon miR‐17‐5p inhibition (Fig. 2A). Similarly, inhibition of miR‐19a‐3p in H727 cells lead to a significant increase of *ALG2* and a slight nonsignificant trend of *REEP3* levels (Fig. 2A). In BON‐1 cell lines, a nonsignificant reduction of miR‐19a‐3p was observed upon inhibitor transfection which led to a significant *ALG2* increase and a nonsignificant increase trend for *REEP3* (Fig. 2A). In addition, *in silico* validation of the 28 prognostic target genes was performed using publicly available data. Similar positive and negative correlations between the miRNAs and their target genes as those documented in our NEN cohort were observed in seven TCGA cohorts, further supporting the miRNA/target gene functional interactions (Fig. 2B).

### 3.5 An 8‐miRNA signature stratifies prognosis of NEN patients

**Fig. 3:**
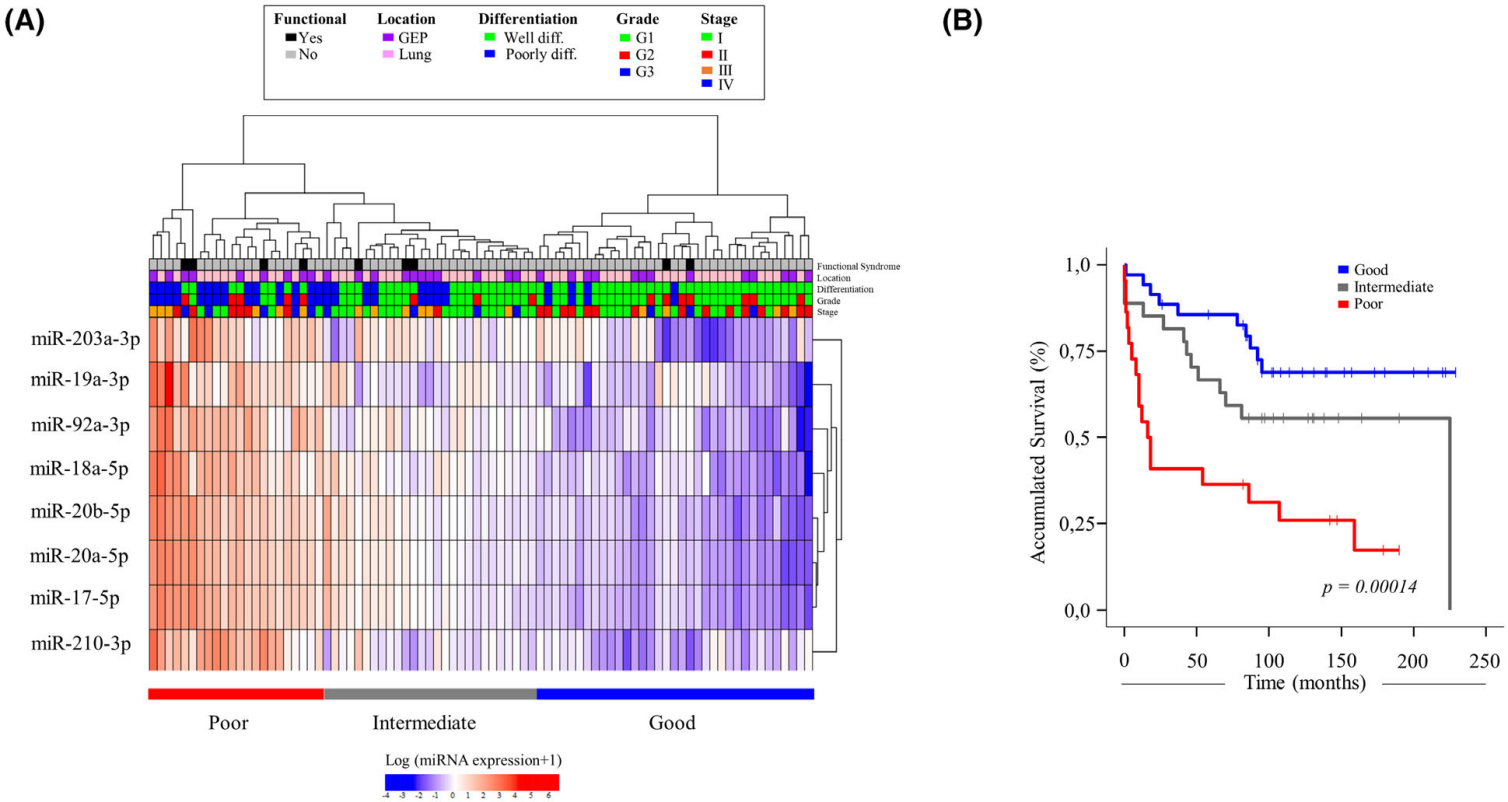
8‐miRNA signature defines prognostic groups of NEN patients. (A) We conducted a heatmap using the expression profile of the eight miRNAs in tumour samples of the NEN patient cohort. Red and blue colours represent high and low expression, respectively. Hierarchical clustering was performed for patients (columns) and miRNAs (rows). The upper tree shows three clusters defined by the 8‐miRNA signature expression profile and survival analysis: poor (left), intermediate (middle) and good prognosis (right). Clustering based on the expression of miRNAs is shown in the tree on the right. Patients were annotated with primary tumour site, grade, stage, differentiation and the presence or absence of a functional syndrome. diff, differentiated; G1, grade 1; G2, grade 2; G3, grade 3; (B) Kaplan–Meier curves of NEN patient prognostic clusters according to the 8‐miRNA expression profile identified three distinct prognostic groups, with 5‐year survival rates of 80% (good prognosis, depicted in blue), 66% (intermediate prognosis, depicted in grey) and 36% (poor prognosis, depicted in red) (*P* = 0.00014). OS in months is represented on the *x*‐axis and survival probability is shown on the *y*‐axis. Values of *P* < 0.05 were considered statistically significant.

Given the biological and independent prognostic relevance of the eight miRNAs, we defined a signature based on their expression profile. Hierarchical clustering of NEN patients based on the expression levels of the eight miRNAs in the signature permitted the identification of three distinct expression clusters grouped by survival, with 5‐year OS rates of 80% (good prognosis, *N* = 35), 66% (intermediate prognosis, *N* = 27) and 36% (poor prognosis, *N* = 22; *P* < 0.001; Fig. 3). Moreover, Cox multivariate analysis showed that prognostic clusters defined by the 8‐miRNA signature were significantly associated with survival (*P* < 0.05), independently of other relevant clinicopathological variables in NENs such as age, gender, primary tumour location, tumour differentiation and stage (Table 2).

**Table 2:** Multivariate analysis of prognosis clusters defined by the 8‐miRNA signature. Cox multivariate proportional hazards model using OS and the clusters defined by the 8‐miRNA signature as well as other possible confounding variables in 84 NENs is shown. Hazard ratios (HR) and *P*‐values are shown. The clusters defined by the signature were associated with a poorer outcome in NEN patients independently of other relevant variables such as gender, age, primary tumour location, differentiation and stage. CI, confidence interval; Ref, reference.

Variables	HR	CI 95%	Multivariate (*P*‐value)
Clusters “8‐miRNAs signature”			***0.046***
Good prognosis	Ref.	Ref.	–
Intermediate prognosis	1.288	0.519–3.198	*0.585*
Poor prognosis	2.763	1.124–6.792	***0.027***
Sex (female vs. male)	0.465	0.183–1.183	*0.108*
Age	1.047	1.015–1.079	***0.003***
Location (lung vs. GEP)	0.530	0.223–1.205	*0.130*
Differentiation (poorly‐ vs. well‐)	4.803	1.878–12.286	***0.001***
Stage (III–IV vs. I–II)	1.119	0.513–2.445	*0.777*

Bold values stand for significant (*p*< 0.05) values. Italicized value have none significance.

### 3.6 Expression of the eight prognostic miRNAs is regulated at the epigenetic level

**Fig. 4:**
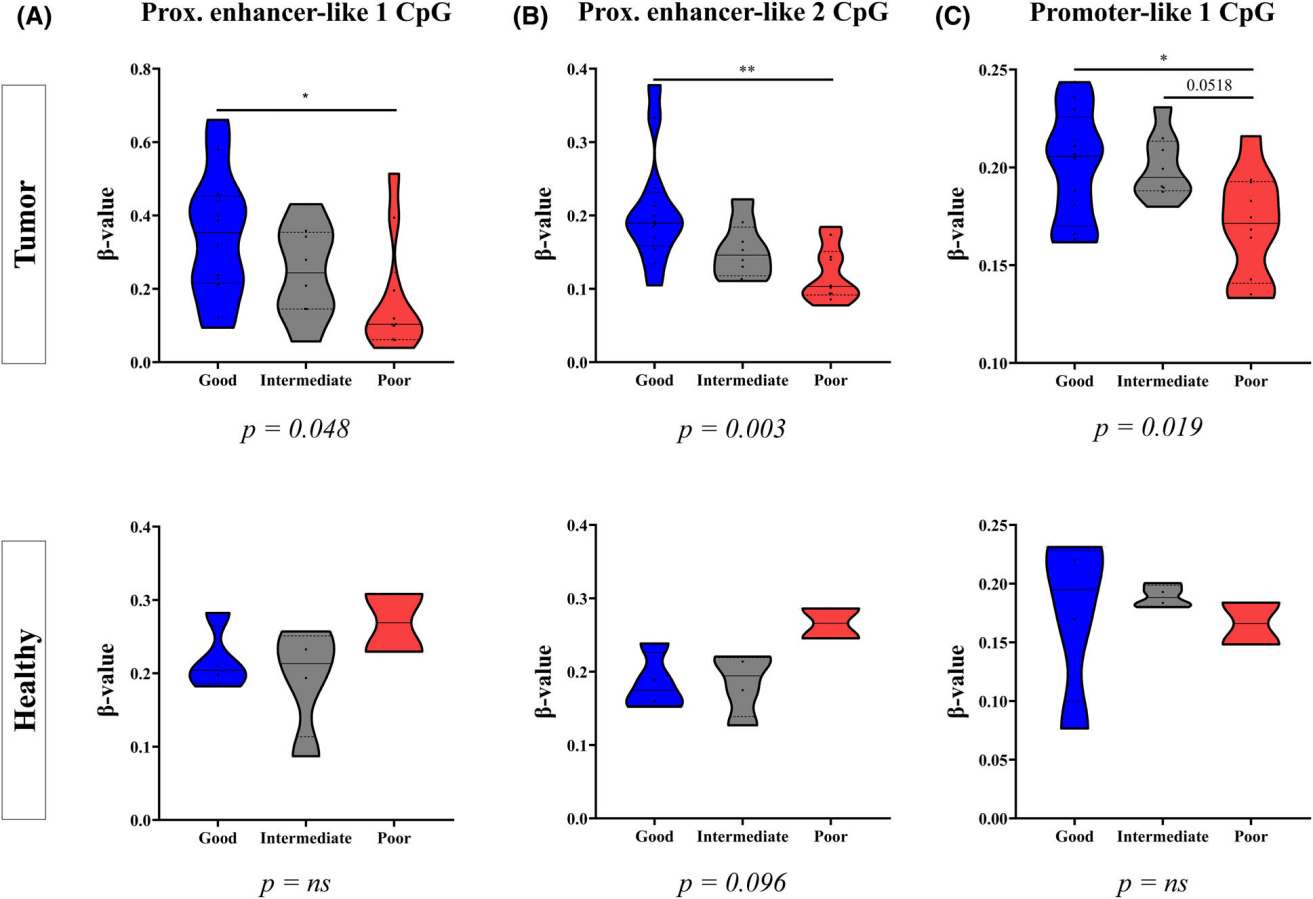
8‐miRNA signature is epigenetically regulated by CpG methylation. Methylation levels (β‐value) of CpG sites which significantly correlated with miRNA expression are shown across the prognostic clusters (defined by miRNA expression profile) in both healthy (*n* = 10) and tumoural tissue (*n* = 30). ANOVA or Kruskall–Wallis test were performed between clusters as appropriate and pairwise comparisons were performed using Tukey's range or Dunn's Test, respectively. (A) The Proximal (Prox.) enhancer‐like 1 CpG site showed no methylation differences in healthy tissue, whereas decreasing methylation was observed for the ‘Good prognosis’ to ‘Poor prognosis’ clusters in tumoural tissue (*P* = 0.048). (B) The Proximal enhancer‐like 2 CpG site showed stable/slightly increasing levels for ‘Good prognosis’ to ‘Poor prognosis’ clusters in paired healthy tissue. Conversely, a significant decrease in methylation was observed from ‘Good prognosis’ to ‘Poor prognosis’ cluster in tumoural tissue (*P* = 0.003). (C) Finally, the Promoter‐like 1 CpG site showed no differences in the normal tissue, and a slight decrease, particularly in the poor prognosis cluster, in the tumoural tissue (*P* = 0.019). *P*‐values <0.05 considered statistically significant (*P < 0.05; **P < 0.01; ns, nonsignificant).

To test whether dysregulation of the 8‐miRNA signature could be mediated by upstream alterations in CpG methylation, we performed DNA methylation analyses on NEN samples (*N* = 30; Table S1) and analysed the correlation between miRNA expression and the methylation levels of CpG sites within 50 kbp of the TSS of each miRNA. Interestingly, the most significantly correlated (FDR < 0.05) CpG sites were located within the *MIR17HG* gene (*oncomiR‐1*), which encodes five of the eight miRNAs of the signature (Table S6). Prox. enhancer‐like CpG 1 (Cg07641807; 91 349 704–91 349 706) and Prox. enhancer‐like CpG 2 (Cg02297838; chr13: 91 350 199–91 350 201) were located in enhancer‐like regions (ENCODE), while Promoter‐like 1 CpG (Cg25308542; chr13: 91 347 858–91 347 860) was located within a CpG island in the *MIR17HG* promoter. Consistently, we observed significantly decreased methylation of these CpG sites in patients from the ‘Poor prognosis’ cluster, which expressed higher levels of the eight miRNAs. Moreover, these methylation differences across prognostic clusters were not observed when we analysed paired healthy tissue (Fig. 4). Accordingly, a robust negative correlation was observed between *MIR17HG* gene expression, and its promoter methylation levels in 8 TCGA cohorts which further supports the epigenetic regulation of *oncomiR‐1* (Fig. S5). Similar observations were made for miR‐210‐3p and two regulatory CpG sites that are involved in its epigenetic regulation (Fig. S6).

## 4 Discussion

Relevant advances in the molecular characterisation of NENs have put the spotlight on epigenetic mechanisms as decisive drivers of these tumours [26, 27]. Although methylation marks and histone modifications are the best known mechanisms of epigenetic regulation, miRNAs are also key molecules involved in the regulation of gene expression [28]. Our study reports a deep characterisation of the expression profile of 84 cancer‐related miRNAs in a cohort of 85 patients with NENs. To this end, we identified for the first time a signature based on the expression profile of eight miRNAs (miR‐17‐5p, ‐18‐5p, ‐19a‐3p, ‐20a‐5p, ‐20b‐5p, ‐92a‐3p, ‐203a‐3p, ‐210‐3p), that is significantly associated with patient prognosis, independent of other well‐established prognostic factors. Specifically, the 8‐miRNA signature permitted the stratification of three distinct prognostic groups, with 5‐year OS rates of 80%, 66% and 36%, respectively (*P* = 0.046). Additional gene expression and DNA methylation analysis for further down‐ and upstream integrative characterisation of dysregulated miRNAs identified key genes and crucial regulatory mechanisms potentially involved in the prognostic outcomes of NEN patients.

Interestingly, five of the eight miRNAs of the signature (miR‐17‐5p, ‐18a‐5p, ‐19a‐3p, ‐20a‐5p and ‐92a‐3p) are members of the miR‐17‐92 cluster (also known as *OncomiR‐1*), localised in chromosome 13, which plays a critical role in cancer [29, 30]. Members of this cluster and paralogs (miR‐20b‐5p) have been found to be involved in angiogenesis, proliferation, autophagy, epithelial–mesenchymal transition, invasiveness, migration, cell cycle and apoptotic processes, and are overexpressed in many haematological and solid tumours. They have also been related to biological aggressiveness and chemotherapy resistance [31]. The downstream molecular mechanisms of miR‐17‐92 are nevertheless poorly understood, although some validated target genes are related to the inhibition of EGR (*early growth response*) [32, 33], *MYC* oncogene or E2F family transcription factors [31], as well as other signalling pathways such as JAK–STAT, RAS/MAPK, PI3K/Akt/mTOR or TGFβ [31, 34].

Knowledge of the role of *oncomiR‐1* in NENs is limited [31, 35]. Coordinated overexpression of miR‐17, ‐20 and ‐92 has been reported in pancreatic acinar and endocrine tumours [36], as well as in angiogenic islets of the RT2 murine model [37]. However, the association of miR‐17‐92 with clinical parameters has not been fully explored. Only miR‐19 overexpression has been related with poorer survival in patients with lung NENs [38]. Our study thus represents the first report of the role of the miR‐17‐92 cluster as an oncogene, associated with a poorer prognosis in NEN patients. Similarly, little is known about the role of miR‐203a in NENs besides its tumour suppressor role in Merkel cell carcinomas [39] and its association with cell growth, migration and invasiveness in other tumours [40, 41]. In the case of miR‐210, Zimmermann et al. [42] described the overexpression of this miRNA in metastasis vs. primary GEP NENs and in samples with higher proliferative rates. Moreover, its role in angiogenesis is also well‐known: miR‐210 levels increases in hypoxia and induce *HIF1A* and *VEGF* expression by inhibiting VEGF's negative regulators or through the JAK/STAT pathway [43].

We also identified in our study a set of 71 target genes, the expression levels of which were significantly correlated (*P* < 0.05) with their respective miRNA. Forty‐one target genes were inversely correlated (*r* < 0), suggesting that their corresponding miRNAs regulate their expression through 3′UTR mRNA binding and degradation. On the contrary, 30 target genes were directly correlated (*r* > 0), which indicates alternative regulation mechanisms. MiRNA/target gene associations were also validated *in silico* using TCGA databases from several solid tumours and NEN cell lines *in vitro*, where we observed upregulation of targets upon miRNA depletion. ORA revealed that these putative targets and, consequently, the miRNAs that regulate them are involved in classic oncogenic processes described in the hallmarks of cancer and in successfully targeted pathways in NEN patients, namely KEGG PI3K‐Akt. Interestingly, epigenetic dysregulation through DNA methylation and miRNA expression modulation could represent a novel mechanism of PI3K‐Akt‐mTOR pathway alterations, which harbours mutations in about 15% of pancreatic NETs and has proven to be a successful target in a wide spectrum of NETs as demonstrated by the mTOR inhibitor Everolimus [44]. This supports the biological relevance of these miRNAs and suggest that miRNA dysregulation could play an important role in driving patients' clinical course.

Finally, we reported here that miR‐17‐92 cluster expression levels are potentially regulated by DNA CpG methylation, which leads to three clusters of patients with different miRNA expression and prognosis. This is in accordance with recent studies pointing to the relevance of epigenetic alterations in NEN development and progression [10, 11, 26, 27, 45]. Interestingly, several authors have shown that the miR‐17‐92 cluster expression can be epigenetically regulated in the context of alveolar differentiation [46, 47, 48]. Moreover, miR‐17‐92 seems to be progressively repressed during lung differentiation, with several studies reporting that miR‐17‐92 cluster overexpression inhibits differentiation, which is consistent with what we have observed in NENs. However, further *in vitro* studies are required to better understand the role of miR‐17‐92 in dedifferentiation [49, 50] and tumour progression, as such efforts could potentially lead to novel therapeutics approaches.

We acknowledge some limitations to our study, including the fact that our cohort was retrospective and heterogeneous in terms of primary tumour site, grade and stage at diagnosis. Nevertheless, the sample size here was not negligible considering the low incidence of the disease, and the patient cohort was well‐characterised from a clinical and pathological perspective. Furthermore, the results obtained were robust, were validated in *in vitro* and *in silico* studies and are biologically plausible as confirmed by integrative transcriptomic and methylomic analyses. Our findings are also supported by preclinical and clinical data published in the scientific literature. Nevertheless, external validation in a prospective independent cohort is warranted to confirm the clinical potential of the described miRNA signature.

## 5 Conclusions

In summary, NENs are mutationally quiet and increasing evidence suggests that epigenetic changes play a major role in NEN development and progression. Our work describes, for the first time, a signature based on the expression levels of eight miRNAs (miR‐17‐5p, ‐18‐5p, ‐19a‐3p, ‐20a‐5p, ‐20b‐5p, ‐92a‐3p, ‐203a‐3p, ‐210‐3p) able to predict survival of patients with GEP and lung NENs, independent of other well‐established prognostic factors. Integrative analyses of miRNA profiles, transcriptomics and methylomics identified key target genes and dysregulated pathways driving prognosis of NEN patients, along with critical CpG sites involved in the epigenetic regulation of these eight miRNAs. Although our findings require external validation in independent series of patients and further functional analyses to better understand the mechanisms by which the eight miRNAs exert their actions in these malignancies, the miRNA signature identified here constitutes a promising tool to facilitate a more accurate prognostic stratification of NEN patients. Furthermore, we have demonstrated that these miRNAs regulate oncogenic pathways and key cellular processes that could potentially be explored in the future for therapeutic purposes.

## Conflict of interest

The authors declare no conflict of interest.

## Author contribution

BS, AL‐P, BA‐P, CC‐P, SM‐P and MF‐F were involved in conceptualisation. BS, AL‐P, PE‐O, CC‐P, SM‐P and LG‐I were involved in methodology. AL‐P and CC‐P were involved in data curation and formal analysis. CC‐P was responsible for software. PE‐O, CC‐P, SM‐P, CR, MB, LG‐I, PM‐B, PJ‐F, YR‐G, AT‐Q, AS, BR and MR were involved in investigation. PE‐O, CR, MB, LG‐I, PM‐B, PJ‐F, YR‐G, AT‐Q and RG‐C were involved in resources. BS and RG‐C were involved in project administration; BS, AL‐P and RG‐C were involved in writing—original draft preparation. BS and RG‐C were involved in reviewing and editing and supervision.

### Peer Review

The peer review history for this article is available at https://publons.com/publon/10.1002/1878‐0261.13393.

## Supporting information


**Fig. S1.** Heatmap showing Spearman correlation among the 84 miRNAs analysed in the whole cohort. All miRNAs are shown in columns and rows. A hierarchical clustering algorithm was performed using a 1‐ Spearman correlation coefficient metric and average as linkage method. Individual correlation coefficient values were colour‐coded, ranging from red (−1, minimum) to blue (+1, maximum). The eight selected miRNAs are shown in red.

**Fig. S2.** Prognostic impact of the 8‐miRNA signature confirmed by qRT‐PCR. (A) Kaplan–Meier curves of the eight prognostic miRNAs in 40 NEN patients from the discovery cohort (N = 40) are shown. Overall survival in months is represented on the x‐axis, whereas survival probability is shown on the y‐axis. The *p‐value* from the *logrank* test using the qRT‐PCR expression data according to high (red) and low (blue) expression by the median is shown in each graph. Four of the eight miRNAs (miR‐17‐5p, miR‐18a‐5p, miR‐20a‐5p and miR‐210‐3p) showed significant prognostic impact. Despite not being statistically significant, miR‐19a‐3p, miR‐20b‐5p and miR‐92a‐3p show a clear trend towards a poorer prognosis with higher miRNA levels. (B) *Cox univariate regression model* data for the 8‐miRNA signature using qPCR expression data as a continuous variable and overall survival. Hazard ratios (*HR*) and *p*‐values are shown. Seven of the eight miRNAs showed a significant prognostic impact on OS, which confirmed our earlier observations with the PCR array. *p* < 0.05 was considered significant.

**Fig. S3.** Functional interactions of 28 prognostic target genes. A string functional network is shown based on the 28 target genes that were significantly associated with OS (*p* < 0.05). Line width increases with higher interaction robustness. Interactions with medium confidence or higher are shown (0.400). Seven genes (*E2F3*, *CCND2*, *E2F8*, *KIF23*, *DUT*, *PRC1* and *RHOB*) are functionally related (PPI enrichment p‐value: 0.0517) despite being regulated by different miRNAs. These genes are involved in mitotic spindle, G2/M checkpoint, apoptosis and cell cycle and proliferation.

**Fig. S4.** Basal miR‐17‐5p and miR‐19a‐3p expression levels in H727 and BON‐1 cell lines are similar to good prognosis NEN patients. MiR‐17‐5p and miR‐19a‐3p expression levels in basal H727 and BON‐1 NET cell lines and in 40 NEN patients from our study which had available RNA were assessed by RT‐PCR. No significant differences in miR‐17‐5p and miR‐19a‐3p levels were observed between NET cell lines and good prognosis NEN patients. Conversely, poor prognosis patients showed a trend towards higher levels of these two miRNAs than NET cell lines (miR‐17‐5p: H727 vs NENs, p = 0.063; BON‐1 vs NENs, p = 0.066; miR‐19a‐3p: H727 vs NENs, p = 0.085; BON‐1 vs NENs, p = 0.088). This is in accordance with the fact that these two cell lines are derived from well‐differentiated NETs, whereas our patient cohort included a wider spectrum of NENs including high grade poorly differentiated NECs. MiRNA expression levels are expressed as 2^−ΔΔCt^ on the y‐axis using H727 cell line as reference. Student's *t‐tests* between the cell lines and the patients from the different prognostic clusters were performed to evaluate differences. Mean ± SEM (Standard Error of the Mean) is shown. *P‐values* <0.05 considered statistically significant.

**Fig. S5.** *MIR17HG* epigenetic correlation is validated in the TCGA. Correlation analysis between *MIR17HG gene* and *MIR17HG* promoter methylation was assessed in eight TCGA studies (Adenocarcinome, ADC; Acute Myeloid Leukemia, AML; Breast Invasive Carcinoma, BRCA; Esophageal Carcinoma, ESCA; Glioblastoma Multiforme, GBM; Kidney Renal Clear Cell Carcinoma, KIRC; Lung Squamous Cell Carcinoma, SLCC; Ovarian.Serous Cystadenocarcinoma, OV). As we observed in our own cohort, *MIR17HG* promoter methylation was inversely correlated with MIR17HG expression.

**Fig. S6.** Significantly correlated CpG sites present differentially methylated levels in NEN tumours. (A) Spearman correlation analysis was performed using PCR‐array miRNA expression and CpG methylation levels of sites within 50 kbp of miR‐210‐3p (regulatory region) in 30 NENs. The Spearman correlation coefficients (r) are shown as well as *p*‐values and adjusted statistical significance (FDR). Promoter‐like 2 and 3 CpG sites (Cg01325426; Chr11:536330–536332 and Cg22174486; Chr11:536816–536818, respectively) are inversely correlated (both FDR =0.07523) with miR‐210‐3p in accordance with the classic CpG methylation regulatory mechanism. These sites are in a CpG island in a promoter‐like region within the *HRAS* gene. (B) The methylation levels (β‐value) of the significantly correlated CpG sites across the 8‐miRNA prognostic clusters were assessed in both healthy and tumoural samples. ANOVA or Kruskall–Wallis test were performed between clusters as appropriate and pairwise comparisons were performed using Tukey's range or Dunn's Test, respectively. Promoter‐like 2 CpG site methylation levels show increasing levels in the three clusters in healthy tissue. Conversely, decreasing levels are observed in the tumoural tissue (*p* = 0.0094). Similar results were obtained for Promoter‐like 3 CpG site, with increasing methylation across clusters in healthy tissue, whereas decreasing methylation is observed in tumoural tissue (*p* = 0.0049).

**Table S1.** Clinico‐pathological features of the study population. The clinic‐pathological characteristic of our study population depicted by the different omic approaches.

**Table S2.** Technical validation of the eight prognostic miRNAs using qRT‐PCR. qRT‐PCR of the eight selected miRNAs was performed on 39 NEN patients in our cohort. A *Spearman* correlation analysis between PCR array and RT‐PCR data (N = 39) for the eight prognostic miRNAs was performed. The *r* value of the *Spearman* correlation coefficient and the *p‐value* are shown. With the exception of miR‐20b‐5p, a strong (r > 0.4) and significant (p < 0.05) correlation was observed between the miRNA array and qRT‐PCR miRNA expression levels for all miRNAs of the signature. *p* < 0.05 was considered significant.

**Table S3.** *In silico* predicted target genes of each selected prognostic miRNA. Genes regulated by more than one miRNA are in bold font.

**Table S4.** List of predicted target genes of the eight selected miRNAs with significant expression correlation with their respective miRNA. *Spearman* correlation coefficients (*r*) using PCR array miRNA and gene expression data of 62 NENs are shown as well as *p*‐values and corresponding miRNA. Predicted target genes are ordered by decreasing *r*. Only predicted target genes with significant correlation are listed in the table (N = 71). Forty‐one of the 71 target genes were inversely correlated (*r* < 0) in accordance with the classical miRNA‐target gene interaction, while 30 showed a direct correlation (*r* > 0), which suggests an alternative regulation. *p* < 0.05 was considered significant. * `Genes that correlate with several miRNAs.

**Table S5.** Overrepresentation analysis (ORA) using the 71 target genes of the eight selected miRNAs and the Hallmarks and KEGG gene sets revealed relevant signalling pathways in NENs. Overrepresentation analysis was performed with *Enrichr* using the 71 target genes that were significantly correlated with their miRNAs, and the gene sets from Hallmarks and KEGG. Gene sets with FDR <0.25 were considered significant. Next, significant pathways, the target genes involved in each pathway and their miRNAs were related (based on their biological functions) to the Hallmarks of cancer which was recently updated by Hanahan and colleagues.

**Table S6.** Significantly correlated CpG sites present differentially methylated levels in NEN tumours.

## Data Availability

The miRNA and mRNA expression and DNA methylation data have been deposited in the Gene Expression Omnibus database under accession number GSE211486. The remaining data are available in this article and its supplementary files or from the authors upon request.
