# Supplementary figures and images for: MicroRNA signature and integrative omics analyses define prognostic clusters and key pathways driving prognosis in patients with neuroendocrine neoplasms
Source: Mol Oncol. 2023 Mar 5;17(4):582–97. doi: 10.1002/1878-0261.13393 (PMC10061291; doi:10.1002/1878-0261.13393)

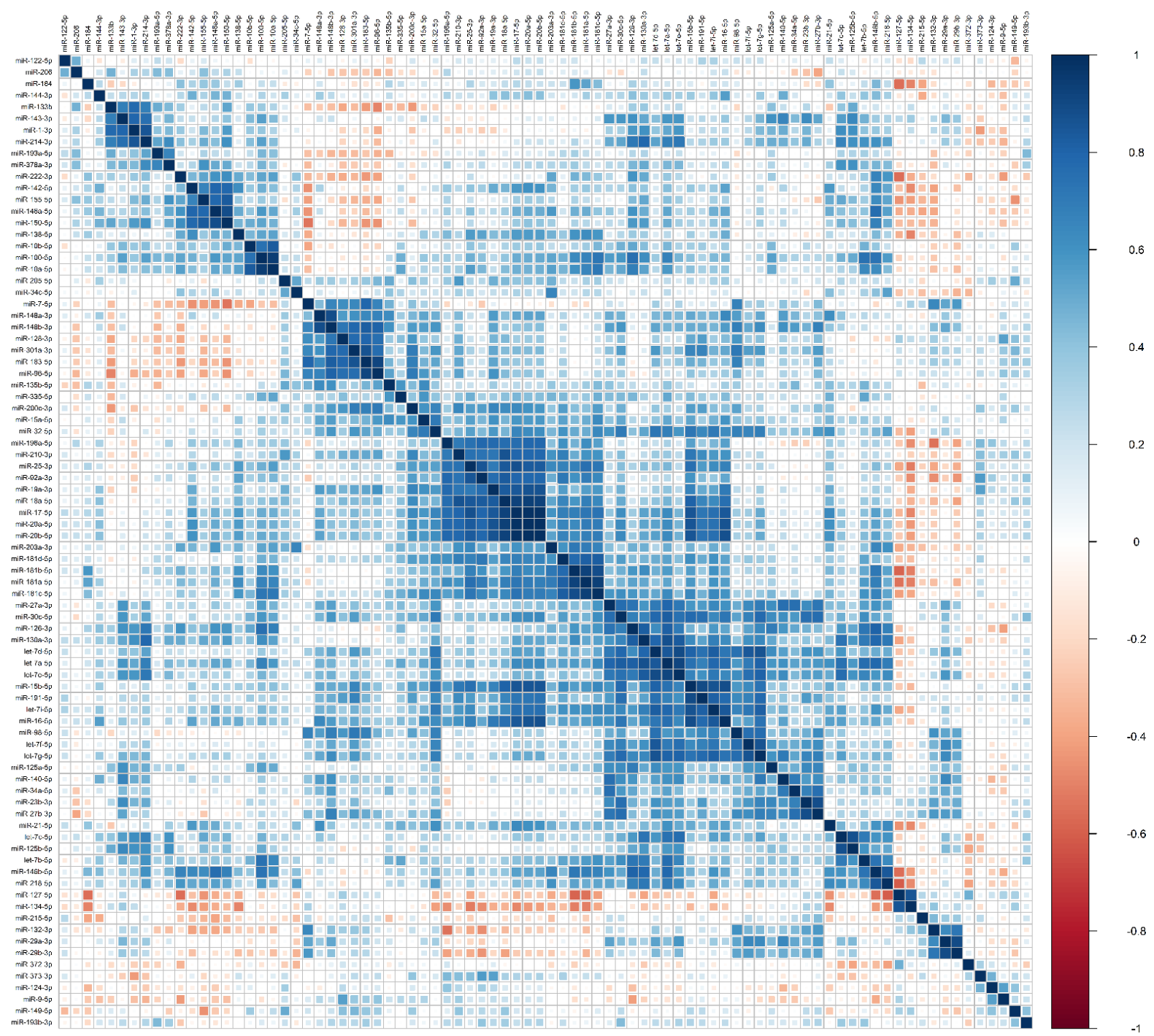

Supplement: Supplementary file 1 — Fig. S1. Heatmap showing Spearman correlation among the 84 miRNAs analysed in the whole cohort. All miRNAs are shown in columns and rows. A hierarchical clustering algorithm was performed using a 1‐ Spearman correlation coefficient metric and average as linkage method. Individual correlation coefficient values were colour‐coded, ranging from red (−1, minimum) to blue (+1, maximum). The eight selected miRNAs are shown in red. [file MOL2-17-582-s006.pdf]

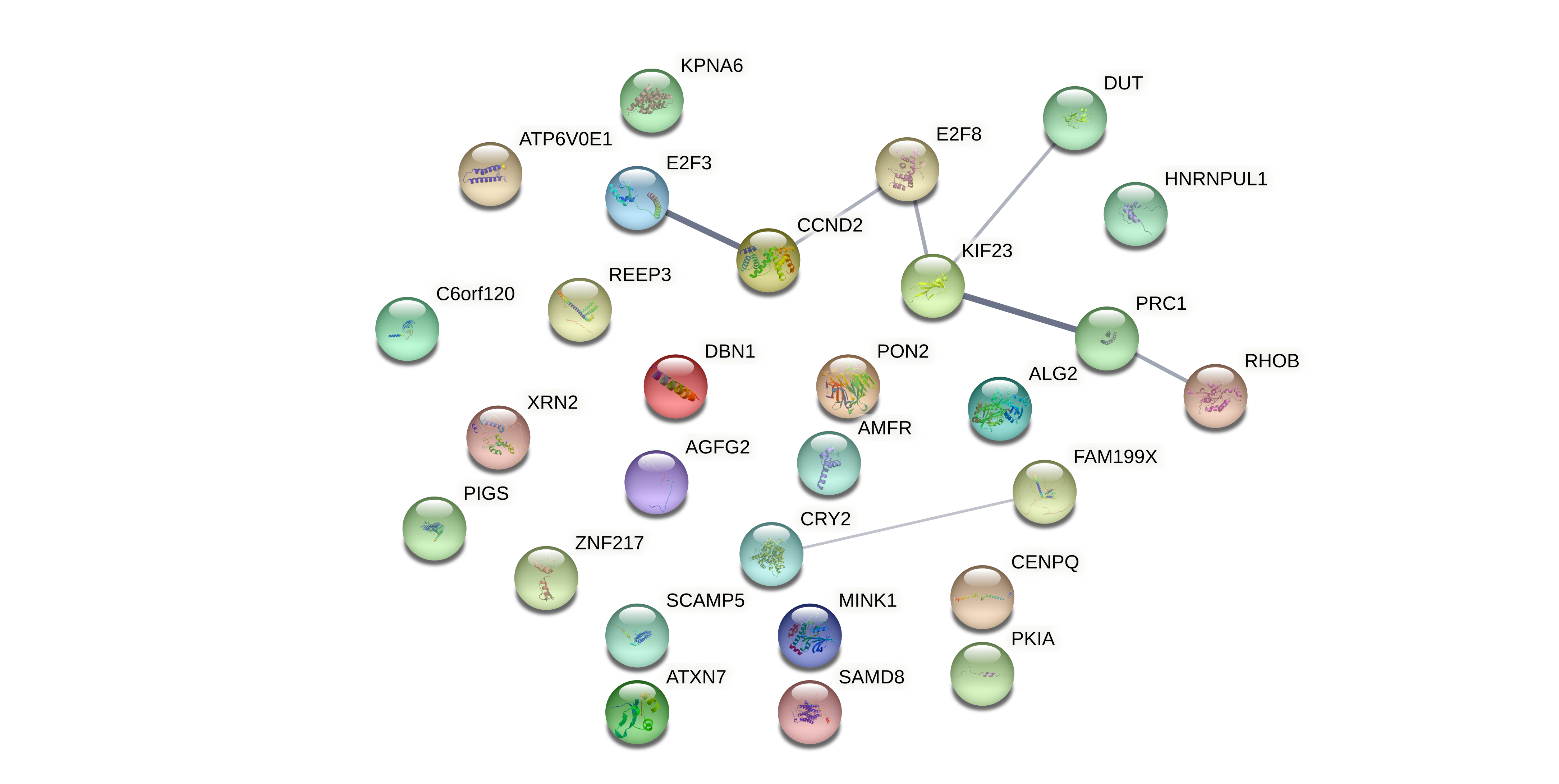

Supplement: Supplementary file 3 — Fig. S3. Functional interactions of 28 prognostic target genes. A string functional network is shown based on the 28 target genes that were significantly associated with OS (p < 0.05). Line width increases with higher interaction robustness. Interactions with medium confidence or higher are shown (0.400). Seven genes (E2F3, CCND2, E2F8, KIF23, DUT, PRC1 and RHOB) are functionally related (PPI enrichment p‐value: 0.0517) despite being regulated by different miRNAs. These genes are involved in mitotic spindle, G2/M checkpoint, apoptosis and cell cycle and proliferation. [file MOL2-17-582-s008.png]

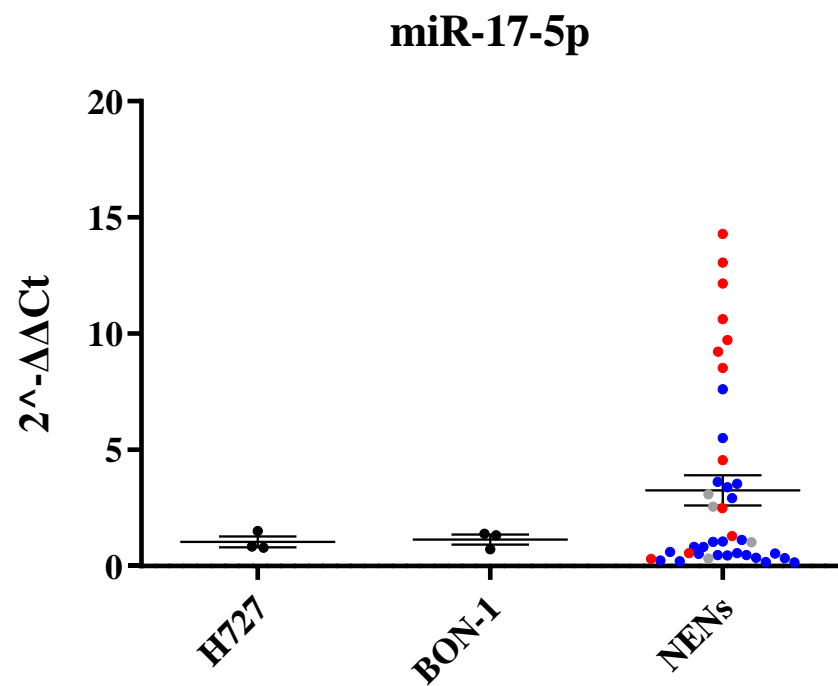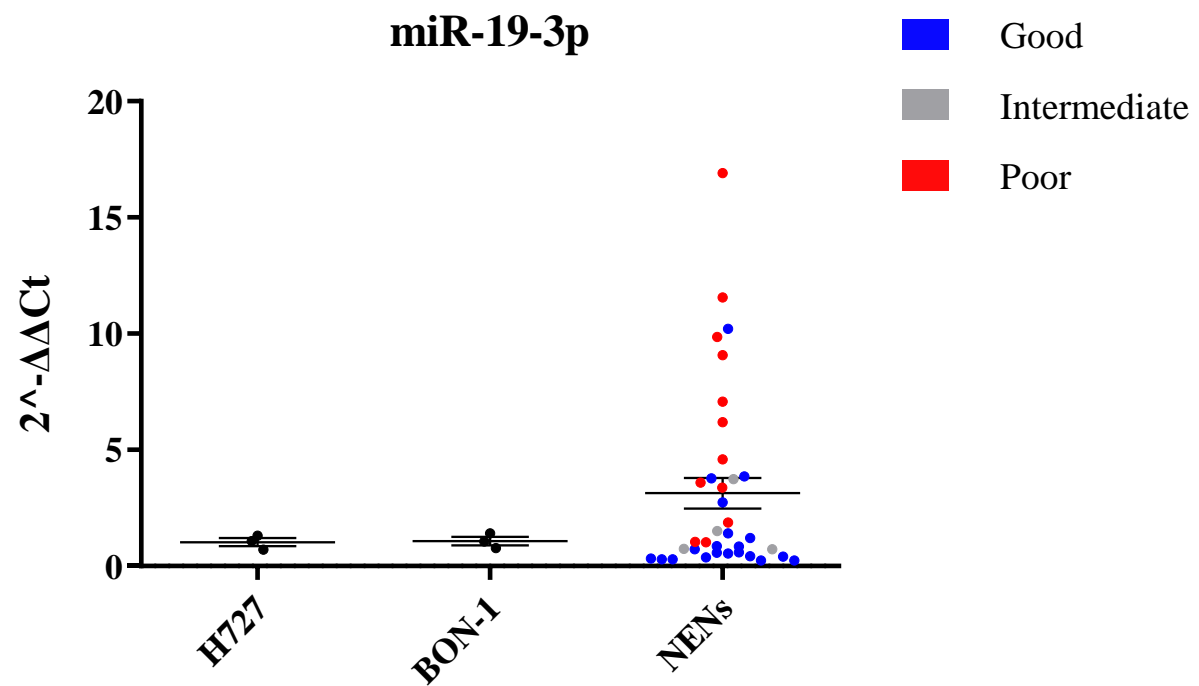

Supplement: Supplementary file 4 — Fig. S4. Basal miR‐17‐5p and miR‐19a‐3p expression levels in H727 and BON‐1 cell lines are similar to good prognosis NEN patients. MiR‐17‐5p and miR‐19a‐3p expression levels in basal H727 and BON‐1 NET cell lines and in 40 NEN patients from our study which had available RNA were assessed by RT‐PCR. No significant differences in miR‐17‐5p and miR‐19a‐3p levels were observed between NET cell lines and good prognosis NEN patients. Conversely, poor prognosis patients showed a trend towards higher levels of these two miRNAs than NET cell lines (miR‐17‐5p: H727 vs NENs, p = 0.063; BON‐1 vs NENs, p = 0.066; miR‐19a‐3p: H727 vs NENs, p = 0.085; BON‐1 vs NENs, p = 0.088). This is in accordance with the fact that these two cell lines are derived from well‐differentiated NETs, whereas our patient cohort included a wider spectrum of NENs including high grade poorly differentiated NECs. MiRNA expression levels are expressed as 2−ΔΔCt on the y‐axis using H727 cell line as reference. Student's t‐tests between the cell lines and the patients from the different prognostic clusters were performed to evaluate differences. Mean ± SEM (Standard Error of the Mean) is shown. P‐values <0.05 considered statistically significant. [file MOL2-17-582-s002.pdf]

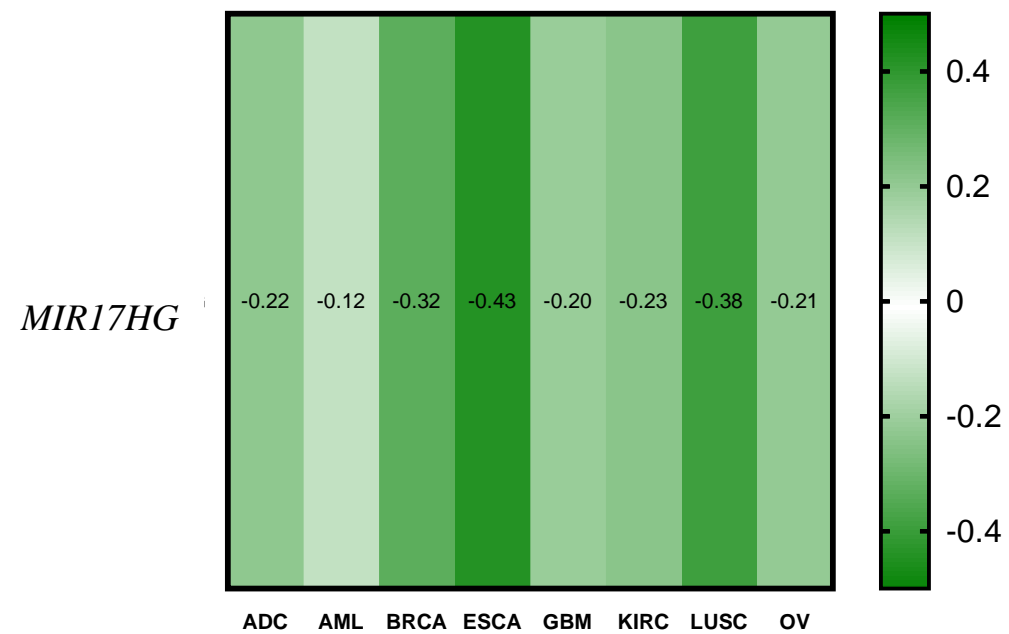

Supplement: Supplementary file 5 — Fig. S5. MIR17HG epigenetic correlation is validated in the TCGA. Correlation analysis between MIR17HG gene and MIR17HG promoter methylation was assessed in eight TCGA studies (Adenocarcinome, ADC; Acute Myeloid Leukemia, AML; Breast Invasive Carcinoma, BRCA; Esophageal Carcinoma, ESCA; Glioblastoma Multiforme, GBM; Kidney Renal Clear Cell Carcinoma, KIRC; Lung Squamous Cell Carcinoma, SLCC; Ovarian.Serous Cystadenocarcinoma, OV). As we observed in our own cohort, MIR17HG promoter methylation was inversely correlated with MIR17HG expression. [file MOL2-17-582-s003.pdf]

A

| MiR-210-3p          | <i>r</i> | <i>P-value</i> | FDR     |
|---------------------|----------|----------------|---------|
| Promoter-like 2 CpG | -0.587   | 0.00065        | 0.07523 |
| Promoter-like 3 CpG | -0.577   | 0.00084        | 0.07523 |

B

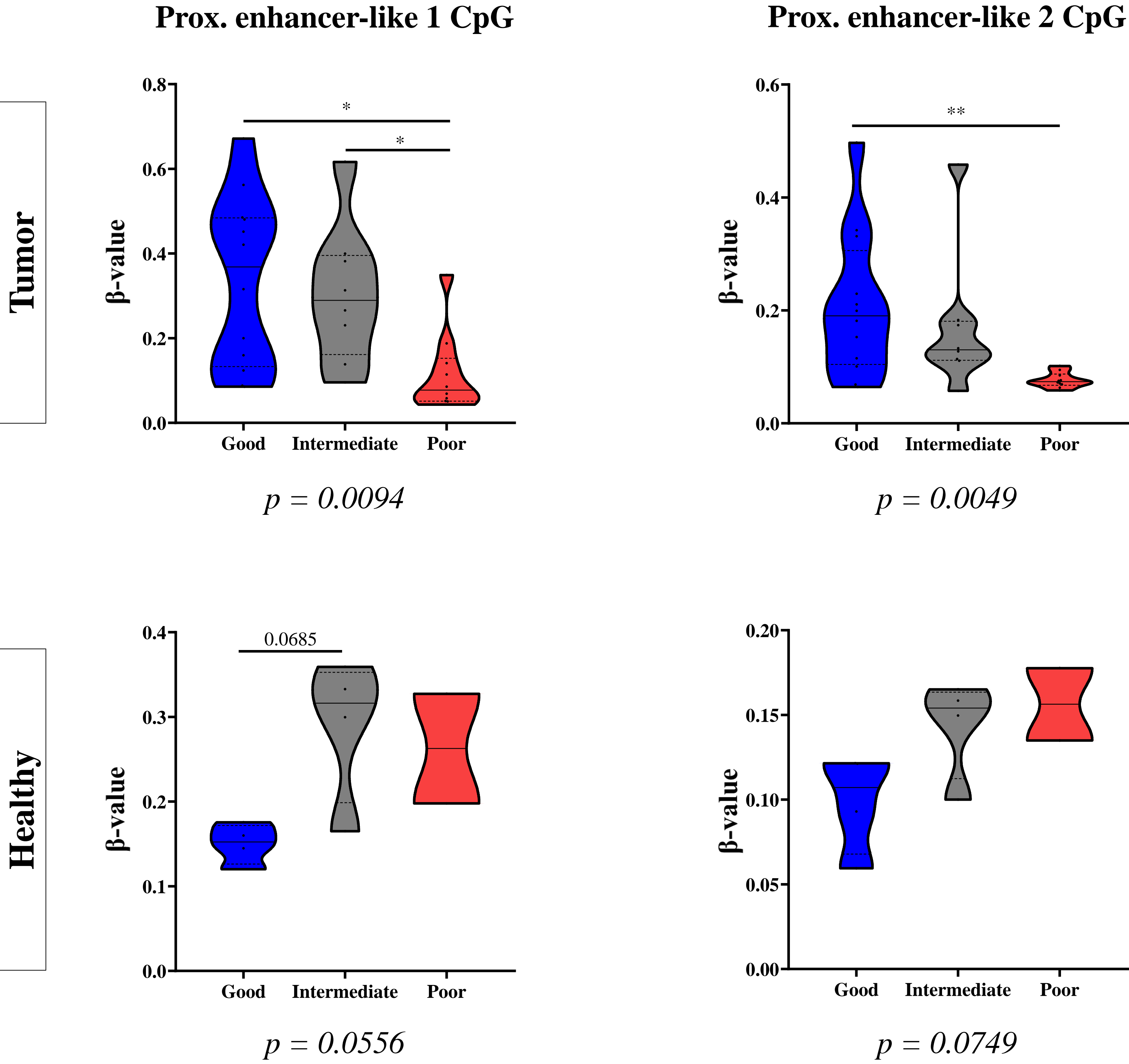

Supplement: Supplementary file 6 — Fig. S6. Significantly correlated CpG sites present differentially methylated levels in NEN tumours. (A) Spearman correlation analysis was performed using PCR‐array miRNA expression and CpG methylation levels of sites within 50 kbp of miR‐210‐3p (regulatory region) in 30 NENs. The Spearman correlation coefficients (r) are shown as well as p‐values and adjusted statistical significance (FDR). Promoter‐like 2 and 3 CpG sites (Cg01325426; Chr11:536330–536332 and Cg22174486; Chr11:536816–536818, respectively) are inversely correlated (both FDR =0.07523) with miR‐210‐3p in accordance with the classic CpG methylation regulatory mechanism. These sites are in a CpG island in a promoter‐like region within the HRAS gene. (B) The methylation levels (β‐value) of the significantly correlated CpG sites across the 8‐miRNA prognostic clusters were assessed in both healthy and tumoural samples. ANOVA or Kruskall–Wallis test were performed between clusters as appropriate and pairwise comparisons were performed using Tukey's range or Dunn's Test, respectively. Promoter‐like 2 CpG site methylation levels show increasing levels in the three clusters in healthy tissue. Conversely, decreasing levels are observed in the tumoural tissue (p = 0.0094). Similar results were obtained for Promoter‐like 3 CpG site, with increasing methylation across clusters in healthy tissue, whereas decreasing methylation is observed in tumoural tissue (p = 0.0049). [file MOL2-17-582-s011.pdf]
