# Supplementary material for: MicroRNA signature and integrative omics analyses define prognostic clusters and key pathways driving prognosis in patients with neuroendocrine neoplasms
Source: Mol Oncol. 2023 Mar 5;17(4):582–97. doi: 10.1002/1878-0261.13393 (PMC10061291; doi:10.1002/1878-0261.13393)
Supplement: Supplementary file 2 — Fig. S2. Prognostic impact of the 8‐miRNA signature confirmed by qRT‐PCR. (A) Kaplan–Meier curves of the eight prognostic miRNAs in 40 NEN patients from the discovery cohort (N = 40) are shown. Overall survival in months is represented on the x‐axis, whereas survival probability is shown on the y‐axis. The p‐value from the logrank test using the qRT‐PCR expression data according to high (red) and low (blue) expression by the median is shown in each graph. Four of the eight miRNAs (miR‐17‐5p, miR‐18a‐5p, miR‐20a‐5p and miR‐210‐3p) showed significant prognostic impact. Despite not being statistically significant, miR‐19a‐3p, miR‐20b‐5p and miR‐92a‐3p show a clear trend towards a poorer prognosis with higher miRNA levels. (B) Cox univariate regression model data for the 8‐miRNA signature using qPCR expression data as a continuous variable and overall survival. Hazard ratios (HR) and p‐values are shown. Seven of the eight miRNAs showed a significant prognostic impact on OS, which confirmed our earlier observations with the PCR array. p < 0.05 was considered significant. [file MOL2-17-582-s010.pdf]

A

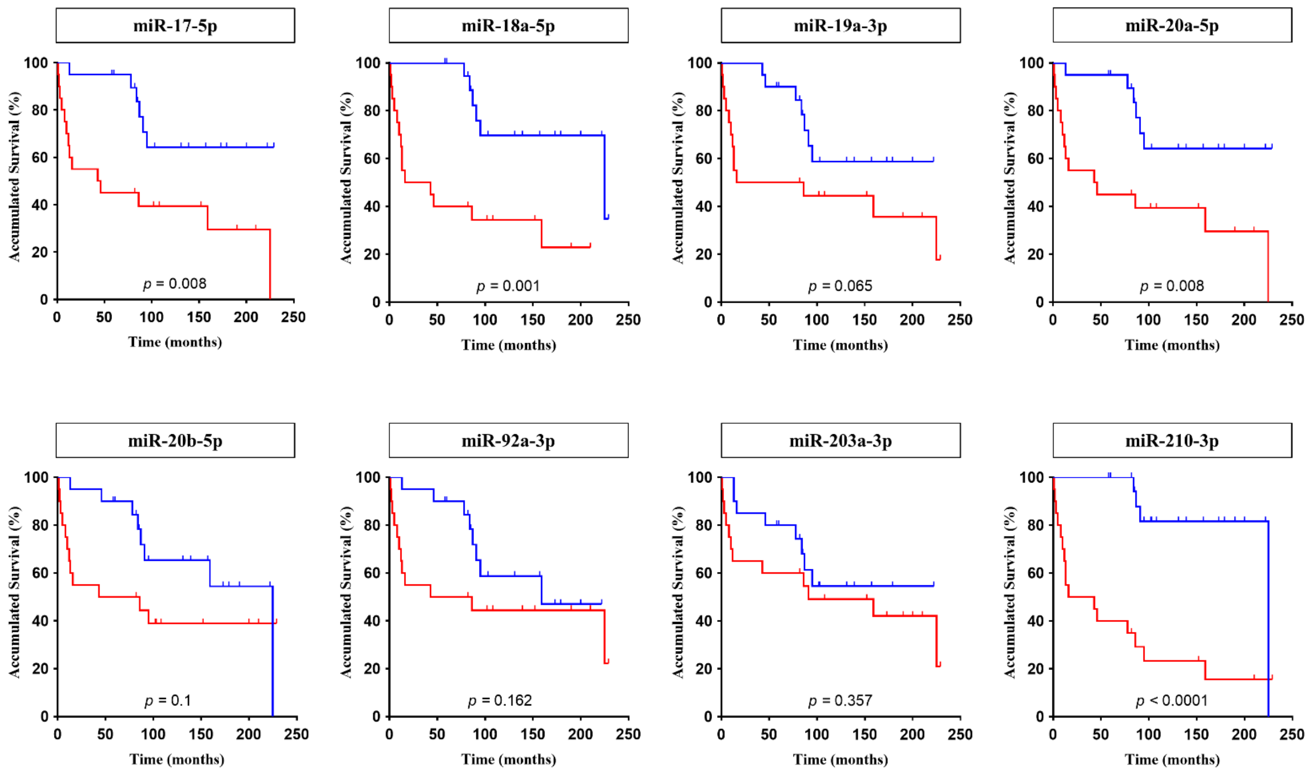

B

Cox regression			
	HR	*(CI 95%)*	*P-value*
miR-17-5p	1.17	(1.07 – 1.27)	***0.00031***
miR-18a-5p	1.04	(1.02 – 1.07)	***0.00082***
miR-19a-3p	1.21	(1.09 – 1.33)	***0.00028***
miR-20a-5p	1.2	(1.07 – 1.34)	***0.0013***
miR-20b-5p	1.22	(1.00 – 1.26)	***0.049***
miR-92a-3p	1.04	(1.01 – 1.07)	***0.0075***
miR-203-3p	0.97	(0.89 – 1.06)	*0.559*
miR-210-3p	1.03	(1.01 – 1.06)	***0.011***
