# Supplementary material for: MicroRNA signature and integrative omics analyses define prognostic clusters and key pathways driving prognosis in patients with neuroendocrine neoplasms
Source: Mol Oncol. 2023 Mar 5;17(4):582–97. doi: 10.1002/1878-0261.13393 (PMC10061291; doi:10.1002/1878-0261.13393)
Supplement: Supplementary file 7 — Table S1. Clinico‐pathological features of the study population. The clinic‐pathological characteristic of our study population depicted by the different omic approaches. [file MOL2-17-582-s001.pdf]

	All (N = 85) N (%)	MiRNAs (N=84) N (%)	Transcriptomic (N=63) N (%)	Methylation (N=30) N (%)	RT-PCR (N=40) N (%)
**Sex**					
Female	37 (43.5)	36 (42.9)	29 (46.0)	14 (46.7)	15 (37.5)
Male	48 (56.5)	48 (57.1)	34 (54.0)	16 (53.3)	25 (62.5)
**Age at diagnosis**					
< 65 years	54 (63.5)	54 (64.3)	41 (65.1)	20 (66.7)	25 (62.5)
≥ 65 years	31 (36.5)	30 (35.7)	22 (34.9)	10 (33.3)	15 (37.5)
**Hormonal syndrome**					
Yes	9 (10.6)	9 (10.7)	4 (6.3)	2 (6.67)	4 (10.0)
No	76 (89.4)	75 (89.3)	59 (93.7)	28 (93.3)	36 (90.0)
**Grade**					
G1/TC	43 (50.6)	43 (51.2)	35 (55.5)	17 (56.6)	18 (45.0)
G2/AC	16 (18.8)	15 (17.9)	12 (19.0)	4 (13.3)	10 (25.0)
G3/LCNEC	26 (30.6)	26 (31.0)	16 (25.4)	9 (30.0)	12 (30.0)
**Histologic differentiation**					
Well-differentiated	59 (69.4)	58 (69.0)	47 (74.6)	21 (70.0)	28 (70.0)
Poorly-differentiated	26 (30.6)	26 (31.0)	16 (25.4)	9 (30.0)	12 (30.0)
**TNM Stage**					
I	34 (40.0)	34 (40.5)	32 (50.8)	18 (60.0)	15 (37.5)
II	22 (25.9)	22 (26.2)	17 (27.0)	6 (20.0)	13 (32.5)
III	16 (18.8)	16 (19.0)	5 (7.9)	3 (10.0)	4 (10.0)
IV	13 (15.3)	12 (14.3)	9 (14.3)	3 (10.0)	8 (20.0)
**Location**					
Lung	51 (60.0)	51 (60.7)	46 (73.0)	25 (83.4)	25 (62.5)
GEP	34 (40.0)	33 (39.3)	17 (27.0)	5 (16.6)	15 (37.5)
**Surgery with radical intent**					
Yes	75 (88.2)	74 (88.1)	57 (90.5)	28 (93.3)	35 (87.5)
No	10 (11.8)	10 (11.9)	6 (9.5)	2 (6.67)	5 (12.5)
**Systemic treatment**					
Somatostatin analogues	18 (21.2)	17 (20.2)	11 (17.5)	4 (13.3)	10 (25.0)
Chemotherapy	17 (20.0)	17 (20.2)	12 (19.0)	9 (30.0)	11 (27.5)
Targeted therapy	10 (11.8)	10 (11.9)	9 (14.3)	2 (6.6)	8 (20.0)
Other	6 (7.1)	6 (7.1)	4 (6.3)	2 (6.6)	4 (10.0)

TC, typical carcinoid; AC, atypical carcinoid; LCNEC, large cell neuroendocrine carcinoma; GEP, gastroenteropancreatic; Targeted Therapy includes: mTOR inhibitors or tyrosine kinase inhibitors.
