# Supplementary material for: MicroRNA signature and integrative omics analyses define prognostic clusters and key pathways driving prognosis in patients with neuroendocrine neoplasms
Source: Mol Oncol. 2023 Mar 5;17(4):582–97. doi: 10.1002/1878-0261.13393 (PMC10061291; doi:10.1002/1878-0261.13393)
Supplement: Supplementary file 8 — Table S2. Technical validation of the eight prognostic miRNAs using qRT‐PCR. qRT‐PCR of the eight selected miRNAs was performed on 39 NEN patients in our cohort. A Spearman correlation analysis between PCR array and RT‐PCR data (N = 39) for the eight prognostic miRNAs was performed. The r value of the Spearman correlation coefficient and the p‐value are shown. With the exception of miR‐20b‐5p, a strong (r > 0.4) and significant (p < 0.05) correlation was observed between the miRNA array and qRT‐PCR miRNA expression levels for all miRNAs of the signature. p < 0.05 was considered significant. [file MOL2-17-582-s004.docx]

|  | **Spearman correlation** | |
| --- | --- | --- |
|  | ***r*** | ***P-value*** |
| miR-17-5p | *0.5656* | ***0.0002*** |
| miR-18a-5p | *0.6915* | ***<0.0001*** |
| miR-19a-3p | *0.4548* | ***0.0036*** |
| miR-20a-5p | *0.5655* | ***0.0002*** |
| miR-20b-5p | *0.3614* | ***0.0238*** |
| miR-92a-3p | *0.6173* | ***<0.0001*** |
| miR-203-3p | *0.5811* | ***0.0001*** |
| miR-210-3p | *0.6233* | ***<0.0001*** |

**Supplementary Table 2. Technical validation of the eight prognostic miRNAs using qRT-PCR.** qRT-PCR of the eight selected miRNAs was performed on 39 NEN patients in our cohort. A *Spearman* correlation analysis between PCR array and RT-PCR data (N=39) for the eight prognostic miRNAs was performed. The *r* value of the *Spearman* correlation coefficient as well as the *p-value* are shown. With the exception of miR-20b-5p, a strong (r >0.4) and significant (p <0.05) correlation was observed between the miRNA array and qRT-PCR miRNA expression levels for all miRNAs of the signature. *p< 0.05* was considered significant.
