# Supplementary material for: MicroRNA signature and integrative omics analyses define prognostic clusters and key pathways driving prognosis in patients with neuroendocrine neoplasms
Source: Mol Oncol. 2023 Mar 5;17(4):582–97. doi: 10.1002/1878-0261.13393 (PMC10061291; doi:10.1002/1878-0261.13393)
Supplement: Supplementary file 10 — Table S4. List of predicted target genes of the eight selected miRNAs with significant expression correlation with their respective miRNA. Spearman correlation coefficients (r) using PCR array miRNA and gene expression data of 62 NENs are shown as well as p‐values and corresponding miRNA. Predicted target genes are ordered by decreasing r. Only predicted target genes with significant correlation are listed in the table (N = 71). Forty‐one of the 71 target genes were inversely correlated (r < 0) in accordance with the classical miRNA‐target gene interaction, while 30 showed a direct correlation (r > 0), which suggests an alternative regulation. p < 0.05 was considered significant. * `Genes that correlate with several miRNAs. [file MOL2-17-582-s007.pdf]

**Gene**	***r***	***P-value***	**MiRNA**
FNDC3A*	-0.452	2.28×10^{-4}	miR-18a-5p
PON2	-0.444	3.40×10^{-4}	miR-20a-5p
REEP3	-0.425	5.74×10^{-4}	miR-19a-3p
AMFR	-0.418	7.16×10^{-4}	miR-203a-3p
CRY2	-0.403	1.16×10^{-3}	miR-17-5p
ALG2	-0.393	1.57×10^{-3}	miR-19a-3p
SMARCA2	-0.368	3.23×10^{-3}	miR-19a-3p
CCND2	-0.354	4.72×10^{-3}	miR-18a-5p
CLOCK	-0.353	4.92×10^{-3}	miR-19a-3p
CRIM1	-0.349	5.45×10^{-3}	miR-18a-5p
TMEM248	-0.345	6.07×10^{-3}	miR-18a-5p
SAR1B	-0.343	6.43×10^{-3}	miR-19a-3p
XYLT2	-0.337	7.45×10^{-3}	miR-18a-5p
SAMD8*	-0.336	7.54×10^{-3}	miR-19a-3p
ZBTB18	-0.320	1.11×10^{-2}	miR-17-5p
RHOB	-0.316	1.24×10^{-2}	miR-19a-3p
TNRC6B	-0.305	1.58×10^{-2}	miR-18a-5p
AGFG2	-0.303	1.69×10^{-2}	miR-17-5p
WAC	-0.301	1.74×10^{-2}	miR-19a-3p
ZER1	-0.300	1.79×10^{-2}	miR-19a-3p
SLC35D1	-0.296	1.94×10^{-2}	miR-19a-3p
FNDC3A*	-0.294	2.04×10^{-2}	miR-19a-3p
CACUL1	-0.287	2.37×10^{-2}	miR-19a-3p
FAM199X	-0.285	2.52×10^{-2}	miR-20a-5p
KLF10	-0.284	2.55×10^{-2}	miR-19a-3p
ATG2B	-0.283	2.56×10^{-2}	miR-17-5p
KPNA6	-0.283	2.57×10^{-2}	miR-18a-5p
SAMD8*	-0.283	2.60×10^{-2}	miR-20b-5p
NR2C2	-0.282	2.69×10^{-2}	miR-20a-5p
PDE4D	-0.275	3.06×10^{-2}	miR-18a-5p
PIGS	-0.275	3.07×10^{-2}	miR-19a-3p
ATXN7	-0.272	3.22×10^{-2}	miR-92a-3p

WDR44	-0.271	3.30×10^{-2}	miR-19a-3p
AFTPH	-0.268	3.49×10^{-2}	miR-19a-3p
HCFC2	-0.268	3.52×10^{-2}	miR-19a-3p
MRPL35	-0.267	3.57×10^{-2}	miR-18a-5p
MINK1	-0.263	3.86×10^{-2}	miR-17-5p
SCAMP5	-0.261	4.05×10^{-2}	miR-20a-5p
SAR1A	-0.260	4.09×10^{-2}	miR-18a-5p
FOXP2	-0.260	4.13×10^{-2}	miR-19a-3p
NBEA	-0.257	4.39×10^{-2}	miR-203a-3p
VPS37A	-0.254	4.63×10^{-2}	miR-19a-3p
ATP6V0E1	-0.253	4.71×10^{-2}	miR-19a-3p
KIAA1191	0.251	4.88×10^{-2}	miR-17-5p
BCL2L11	0.253	4.70×10^{-2}	miR-203a-3p
PMEPA1	0.259	4.21×10^{-2}	miR-19a-3p
YWHAQ	0.261	4.01×10^{-2}	miR-203a-3p
AZIN1	0.263	3.87×10^{-2}	miR-203a-3p
ZNF217	0.264	3.79×10^{-2}	miR-19a-3p
UBXN2A	0.274	3.14×10^{-2}	miR-20a-5p
C6orf120	0.277	2.92×10^{-2}	miR-17-5p
E2F3	0.282	2.66×10^{-2}	miR-17-5p
DBN1	0.282	2.61×10^{-2}	miR-19a-3p
PKIA	0.284	2.54×10^{-2}	miR-20a-5p
HPRT1	0.290	2.24×10^{-2}	miR-19a-3p
CENPQ	0.292	2.11×10^{-2}	miR-17-5p
ARMC8	0.294	2.03×10^{-2}	miR-19a-3p
LSM14B	0.300	1.79×10^{-2}	miR-18a-5p
LAMP2	0.301	1.76×10^{-2}	miR-203a-3p
PRC1	0.302	1.70×10^{-2}	miR-19a-3p
SLC40A1	0.307	1.55×10^{-2}	miR-20a-5p
PTPRD	0.312	1.38×10^{-2}	miR-20a-5p
HNRNPUL1	0.314	1.28×10^{-2}	miR-19a-3p
E2F8	0.321	1.10×10^{-2}	miR-19a-3p
RAF1	0.322	1.08×10^{-2}	miR-19a-3p
XRN2	0.326	9.81×10^{-3}	miR-203a-3p

CA12	0.339	6.97×10^{-3}	miR-18a-5p
KIF23	0.343	6.33×10^{-3}	miR-20b-5p
DUSP7	0.350	5.35×10^{-3}	miR-19a-3p
ELK4	0.358	4.31×10^{-3}	miR-203a-3p
LDHA	0.416	7.80×10^{-4}	miR-203a-3p
DUT	0.429	5.11×10^{-4}	miR-19a-3p
PHF19	0.443	3.17×10^{-4}	miR-18a-5p
