# Supplementary material for: MicroRNA signature and integrative omics analyses define prognostic clusters and key pathways driving prognosis in patients with neuroendocrine neoplasms
Source: Mol Oncol. 2023 Mar 5;17(4):582–97. doi: 10.1002/1878-0261.13393 (PMC10061291; doi:10.1002/1878-0261.13393)
Supplement: Supplementary file 11 — Table S5. Overrepresentation analysis (ORA) using the 71 target genes of the eight selected miRNAs and the Hallmarks and KEGG gene sets revealed relevant signalling pathways in NENs. Overrepresentation analysis was performed with Enrichr using the 71 target genes that were significantly correlated with their miRNAs, and the gene sets from Hallmarks and KEGG. Gene sets with FDR <0.25 were considered significant. Next, significant pathways, the target genes involved in each pathway and their miRNAs were related (based on their biological functions) to the Hallmarks of cancer which was recently updated by Hanahan and colleagues. [file MOL2-17-582-s005.pdf]

Hallmark	P-value	FDR	Odds Ratio	Combined Score	Genes	miRNAs
**Sustaining proliferative signals**						
Hallmarks Myc Targets V1	0.005	0.099	6.01	31.17	LDHA, DUT, YWHAQ, HPRT1	-19a, -203a
KEGG cell cycle	0.009	0.197	7.22	33.42	CCND2, YWHAQ, E2F3	-17, -18a, -203a
KEGG PI3K-Akt signalling pathway	0.037	0.215	3.34	11.0	CCND2,YWHAQ BCL2L11, RAF1	-18a,-19a, -203a
Hallmark TNFα Sig. via NF-kB	0.034	0.099	4.41	14.91	KLF10, PMEPA1, RHOB	-19a
**Avoiding growth suppression**						
Hallmarks Myc Targets V1	0.005	0.099	6.01	31.17	LDHA, DUT, YWHAQ, HPRT1	-19a, -203a
KEGG FoxO signalling pathway	0.011	0.187	6.82	30.56	CCND2, BCL2L11, RAF1	-18a, -19a, -203a
Hallmark TGF-beta Signalling	0.015	0.099	11.08	45.97	KLF10, PMEPA1	-19a
**Epigenetic reprogramming**						
Hallmarks OXPHOS	0.034	0.099	4.42	14.91	LDHA, ATP6V0E1, MRPL35	-203a-, -19a, -18a
**Avoiding immune destruction**						
Hallmarks Myc Targets V1	0.005	0.099	6.01	31.17	LDHA, DUT, YWHAQ, HPRT1	-19a, -203a
Hallmark TGF-beta Signalling	0.015	0.099	11.08	45.97	KLF10, PMEPA1	-19a
**Enabling replicative immortality**						
**Tumour promoting inflammation**						
**Polymorphic microbiomes**						
**Activating invasion and metastasis**						
Hallmarks Myc Targets V1	0.005	0.099	6.01	31.17	LDHA, DUT, YWHAQ, HPRT1	-19a, -203a
Hallmark TNFα Sig. via NF-kB	0.034	0.099	4.41	14.91	KLF10, PMEPA1, RHOB	-19a
Hallmark TGF-beta Signalling	0.015	0.099	11.08	45.97	KLF10, PMEPA1	-19a
**Inducing angiogenesis**						
Hallmarks Myc Targets V1	0.005	0.099	6.01	31.17	LDHA, DUT, YWHAQ, HPRT1	-19a, -203a
Hallmark TNFα Sig. via NF-kB	0.034	0.099	4.41	14.91	KLF10, PMEPA1, RHOB	-19a
Hallmarks OXPHOS	0.034	0.099	4.42	14.91	LDHA, ATP6V0E1, MRPL35	-203a-, -19a, -18a
**Senescent cells**						

KEGG Cellular senescence	0.018	0.197	5.70	22.88	CCND2, E2F3, RAF1	-17, -18a, -19a
**Genome instability and mutations**						
Hallmarks Mitotic Spindle	0.033	0.099	4.44	15.04	PRC1, KIF23, BCL2L11	-19a, -20b, -203a
Hallmarks Myc Targets V1	0.005	0.099	6.01	31.17	LDHA, DUT, YWHAQ, HPRT1	-19a, -203a
KEGG FoxO signalling pathway	0.011	0.187	5.70	30.56	CCND2, BCL2L11, RAF1	-18a, -19a, -203a
Hallmarks UV Response Up	0.018	0.099	5.63	22.40	PTPRD, BCL2L11, RHOB	-19a, -20a, -203a
Hallmarks G2-M Checkpoint	0.034	0.125	4.42	14.91	PRC1, KIF23, E2F3	-17, -19a, -20b
**Resisting cell death**						
Hallmarks Myc Targets V1	0.005	0.099	6.01	31.17	LDHA, DUT, YWHAQ, HPRT1	-19a, -203a
KEGG FoxO signalling pathway	0.011	0.187	6.82	30.56	CCND2, BCL2L11, RAF1	-18a, -19a, -203a
KEGG Autophagy	0.012	0.197	6.52	28.40	LAMP2, RAF1, ATG2B	-17, -19a, -203a
Hallmarks Apoptosis	0.019	0.099	5.52	21.70	CCND2, BCL2L11, RHOB	-18a, -19a, -203a
Hallmarks G2-M Checkpoint	0.034	0.099	4.42	14.91	PRC1, KIF23, E2F3	-17, -19a, -20b
Hallmark TGF-beta Signalling	0.015	0.099	11.08	45.97	KLF10, PMEPA1	-19a
**Deregulating cellular energetics**						
KEGG FoxO signalling pathway	0.011	0.187	6.82	30.56	CCND2, BCL2L11, RAF1	-18a, -19a, -203a
KEGG Central carbon met. in cancer	0.025	0.197	8.47	31.00	LDHA, RAF1	-19a, -203a
Hallmarks OXPHOS	0.034	0.099	4.42	14.91	LDHA, ATP6V0E1, MRPL35	-203a-, -19a, -18a
**Unlocking phenotypic plasticity**						
KEGG PI3K-Akt signalling pathway	0.037	0.215	3.34	11.0	CCND2,YWHAQ BCL2L11, RAF1	-18a, -19a, -203a
