# Supplementary material for: MicroRNA signature and integrative omics analyses define prognostic clusters and key pathways driving prognosis in patients with neuroendocrine neoplasms
Source: Mol Oncol. 2023 Mar 5;17(4):582–97. doi: 10.1002/1878-0261.13393 (PMC10061291; doi:10.1002/1878-0261.13393)
Supplement: Supplementary file 12 — Table S6. Significantly correlated CpG sites present differentially methylated levels in NEN tumours. [file MOL2-17-582-s009.pdf]

A

**Prox. enhancer-like 1**	***r***	***P-value***	**FDR**
**miR-17-5p**	-0.527	0.00278	0.03750
**miR-18a-5p**	-0.659	0.00011	0.00202
**miR-19a-3p**	-0.557	0.00138	0.01279
**miR-20a-5p**	-0.507	0.00473	0.06385
**miR-92a-3p**	-0.621	0.00025	0.00685

B

**Prox. enhancer-like 2**	***r***	***P-value***	**FDR**
**miR-17-5p**	-0.640	0.00014	0.00376
**miR-18a-5p**	-0.649	0.00015	0.00202
**miR-19a-3p**	-0.577	0.00085	0.01279
**miR-20a-5p**	-0.644	0.00018	0.00474
**miR-92a-3p**	-0.594	0.00053	0.00722

C

**Promoter-like 1**	***r***	***P-value***	**FDR**
**miR-17-5p**	-0.469	0.00901	0.08109
**miR-18a-5p**	-0.499	0.00552	0.04972
**miR-19a-3p**	-0.556	0.00142	0.01279
**miR-20a-5p**	-0.474	0.00884	0.07959
miR-92a-3p	-0.348	0.05882	0.46724
